# Rational Design and Lead Optimisation of Potent Antimalarial Quinazolinediones and Their Cytotoxicity against MCF-7

**DOI:** 10.3390/molecules28072999

**Published:** 2023-03-28

**Authors:** Sitthivut Charoensutthivarakul, Duangporn Lohawittayanan, Phongthon Kanjanasirirat, Kedchin Jearawuttanakul, Sawinee Seemakhan, Napason Chabang, Patrick Schlaeppi, Varisa Tantivess, Tanapol Limboonreung, Matthew Phanchana

**Affiliations:** 1 Innovative Molecular Discovery Laboratory (iMoD), School of Bioinnovation and Bio-Based Product Intelligence, Faculty of Science, Mahidol University, Bangkok 10400, Thailand; 2 Excellent Center for Drug Discovery, Faculty of Science, Mahidol University, Bangkok 10400, Thailand; 3 Center for Neuroscience, Faculty of Science, Mahidol University, Bangkok 10400, Thailand; 4 School of Dentistry, King Mongkut’s Institute of Technology Ladkrabang, Bangkok 10520, Thailand; 5 Department of Molecular Tropical Medicine and Genetics, Faculty of Tropical Medicine, Mahidol University, Bangkok 10400, Thailand

**Keywords:** quinazolinedione derivatives, antimalarial activity, antiproliferative activity

## Abstract

Quinazolinedione is one of the most outstanding heterocycles in medicinal chemistry thanks to its wide ranges of biological activities including antimalarial, anticancer, and anti-inflammatory. TCMDC-125133 containing a quinazolinedione pharmacophore displays promising antimalarial activity and low toxicity, as described in the GlaxoSmithKline (GSK) report. Herein, the design and synthesis of novel quinazolinedione derivatives is described on the basis of our previous work on the synthesis of TCMDC-125133, where low-cost chemicals and greener alternatives were used when possible. The initial SAR study focused on the replacement of the valine linker moiety; according to the in silico prediction using SwissADME, concise four-step syntheses toward compounds **4**–**10** were developed. The in-house synthesized compounds **4**–**10** were assayed for antimalarial activity against *P. falciparum* 3D7, and the result revealed that only the compound **2** containing a valine linker was tolerated. Another round of lead optimization focused on the replacement of the *m*-anisidine moiety in compound **2**. A library of 12 derivatives was prepared, and the antimalarial assay showed that potent antimalarial activity could be maintained by replacing the methoxy group in the meta position of the phenyl side chain with a fluorine or chlorine atom (**21**: IC_50_ = 36 ± 5 nM, **24**: IC_50_ = 22 ± 5 nM). Further lead optimization is underway to enhance the antimalarial activity of this class of compound. The compounds included in the study possess little to no antiproliferative activity against MCF-7 cells.

## 1. Introduction

Malaria is a health challenge with around 247 million cases and more than 619,000 deaths in 2021, mostly in sub-Saharan Africa, as reported in the WHO’s 2022 malaria report [1]. The disease is caused by *Plasmodium* parasites, of which *P. falciparum* is the deadliest form; it is then transmitted to human by *Anopheles* mosquito vectors. Artemisinin-based combination therapies (ACTs) have been employed as the front-line treatment over the past decade in tackling global malaria deaths; however, there are several reports demonstrating that *P. falciparum* has developed resistance to this class of therapy [2,3,4,5]. Therefore, the discovery and development of novel antimalarial chemotypes with novel modes of action are urgently required to circumvent cross-resistance with existing drugs. The discovery of novel drug candidates focusing on new chemical scaffolds that have never been explored for their antimalarial activity remains a top priority to back up current ACTs.

**Figure 1:**
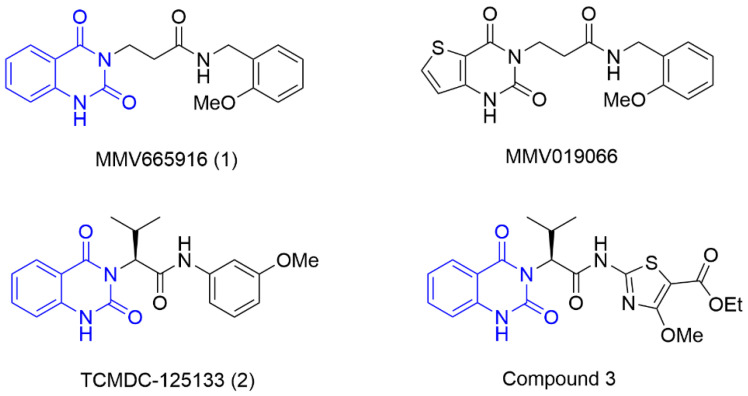
Structures of MMV665916, MMV019066, TCMDC-125133, and compound **3** with their quinazolinedione moiety highlighted in blue.

During the past decade, many research groups have worked on antimalarial drug discovery initiatives, and the structures of thousands of lead compounds have been published in the public domain, allowing other researchers to work on those starting points [6,7]. Amongst them, the Tres Cantos Antimalarial Set (TCAMS) containing thousands of antimalarial hits is one of the largest set of potential molecules ever published in the antimalarial drug discovery community [8]. In the TCAMS initiative, high-throughput phenotypic screening against asexual stage *P. falciparum* was set up with access to the GSK corporate library of over one million compounds. The hits from these screenings were analyzed and prioritized according to their favorable characteristics [8]. One of the highly potent molecules identified as a singleton hit from the TCAMS screening is TCMDC-125133 (**2**). This hit compound features a quinazolinedione pharmacophore with valinyl side chain (Figure 1).

Quinazolinedione is a commonly present heterocycle in pharmaceutical and bioactive molecules that possess a broad spectrum of biological activities, including antimalarial, anticancer, antihypertensive, antiviral, and anti-inflammatory [9,10,11,12,13]. Many quinazolinedione derivatives have been recently reported to possess potent antimalarial activity. Quinazolinedione-based MMV665916 (**1**) and MMV019066 (Figure 1) demonstrated potent parasite growth inhibitory activity against multiple stages of the malaria parasite (<1 µM against *P. falciparum* strains), as reported by TCAMS [8,14,15]. In our group, we recently reported the synthesis of antimalarial quinazolinedione **2** with an IC_50_ of around 200 nM against *P. falciparum* 3D7 [16]. This compound was also assayed for antiproliferative activity against breast cancer cell line MCF-7 and showed mild inhibitory activity with an IC_50_ of 17.5 µM [16]. 

Breast cancer is one of the most common types of cancer. Most women diagnosed with breast cancer are over the age of 50, with around one in eight women diagnosed with this cancer during their lifetime [17]. In 2018, this cancer led to two million new cases and over 627,000 deaths [18]. It is well evident that quinazolinedione derivatives possess anticancer activity. Akgun et al. reported the synthesis of quinazolinedione derivatives and their antiproliferative activities against three different cancer cell lines, and the results showed that some derivatives exhibited cytotoxicity below 10 µM [19]. Compound **3** (Figure 1) and related compounds, which are 3-substituted-2,4-quinazolinedione derivatives, are patented for their highly potent cytotoxicity against human ovarian cancer (SKOV3) [20]. 

To further progress the quinazolinedione **2** in an antimalarial drug discovery pipeline, it is necessary to understand the structure–activity relationships (SARs) around this pharmacophore. Therefore, in this paper, the proof-of-concept lead optimization was established using a rational design approach around the side chain of quinazolinedione **2**. Rapid and rational exploration of SARs was made possible thanks to the use of web-based SwissADME in silico prediction [21]. A total of 19 final compounds were synthesized, and their SARs against the *P. falciparum* 3D7 strain were explored, along with their cytotoxicity against MCF-7 cells.

**Figure 2:**
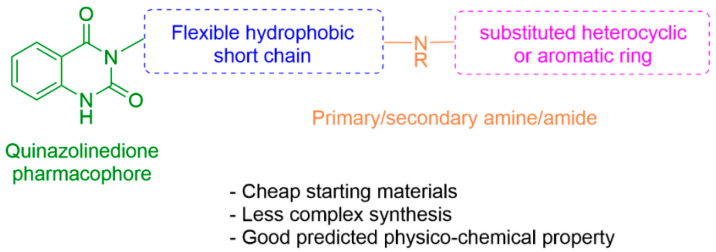
Medicinal chemistry rationale to probe the SARs around the quinazolinedione core.

The crucial part of this work was the design and synthesis of quinazolinedione derivatives that contain various functionalities and side chains as outlined in Figure 2. Their antimalarial activity was assessed against *P. falciparum* in vitro, along with their cytotoxicity against MCF-7 cells. The synthetic strategy involved the incorporation of different hydrophobic side chains to probe the SARs around the valine region, with the aim of increasing the potency to a nanomolar level. As outlined in Figure 2, the chemical synthesis involved the use of commercially available starting materials with a low cost, as this is a crucial target candidate profile (TCP) in the antimalarial drug research community [22]. 

## 2. Results and Discussion

### 2.1. SwissADME In Silico Prediction

To identify potential analogues of quinazolinedione **2** which could be progressed further in a drug discovery pipeline, a preliminary in silico prediction is required. SwissADME, a web-based in silico calculation, is a powerful tool to provide some physicochemical, pharmacokinetic, and ADME (absorption, distribution, metabolism, and excretion) parameters necessary in the small-molecule drug discovery pipeline [21]. SwissADME was used in this work to investigate whether any other hydrophobic short chains would be suitable as a replacement for valine in compound **2**. The valine side chain is a rather bulky hydrophobic moiety and contains an undesirable stereocenter; these factors could hamper further drug development. 

The SMILES strings of compounds **2** and **4**–**10** were entered on the SwissADME website (http://www.swissadme.ch/, accessed on 26 April 2022). The results from the in silico prediction displayed in Table 1 show that other linkers—i.e., aminoethyl (compound **4**), glycine (compound **5**), alanine (compound **6**), and homoalanine (compound **10**)—could be alternatives to valine (compound **2**) as the derivatives containing these linkers (compound **4**–**6** and **10**) showed superior lead-likeness properties, i.e., improved aqueous solubility and better CYP inhibition profiles when compared to the parent compound (compound **2**).

### 2.2. Lead Optimization

From Table 1, we initially prioritized compounds **4**–**6** and **10** due to their synthetic tractability and suitable physicochemical properties including a low logP (lipophilicity), high logS (solubility), and fewer interactions with CYP450s. The first part of this work was to develop a synthetic approach toward all these compounds and assess their antimalarial activity. The syntheses of interest need to match with the scalability and tractability conditions (i.e., robust, compatible with various functionalities, using cheap and commercially available starting materials, and divergent).

**Scheme 1:**
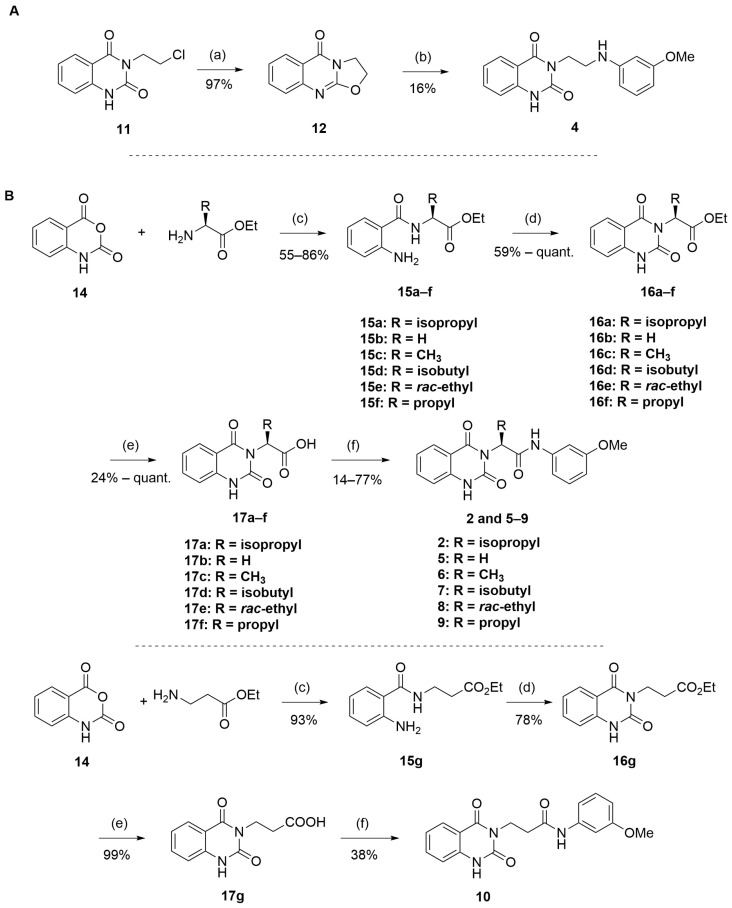
Reagents and conditions: (**a**) K_2_CO_3_, KI (cat.), ACN, 100 °C, overnight; (**b**) *m*-anisidine, ACN, 110 °C, over 2 days; (**c**) K_2_CO_3_, ACN, 60 °C, 18 h; (**d**) CDI, THF, 85 °C, 18 h; (**e**) LiOH, THF/water, 85 °C, 18 h; (**f**) *m*-anisidine, HATU, Et_3_N, DMF, rt, 18 h.

The routes toward these derivatives were successfully developed and identified, and the synthetic plan was divided into two main synthetic routes for two different types of side chains being explored, as depicted in Scheme 1. The first route (A) was designed to produce the quinazolinedione derivatives with an ethyl linker between the core structure and the amine side chain. The synthesis in route A started with the cyclization reaction of commercially available quinazolinedione **11** in acetonitrile (ACN) in the presence of a catalytic amount of KI, and the resultant residues were then purified by flash column chromatography (CC) to yield **12** in 97% yield. The intermediate **12** was then reacted with *m*-anisidine in ACN at 110 °C for 2 days to obtain **4** in 16% yield (see Supplementary Materials for detailed experiment and compound characterization) [23]. 

The synthesis in Scheme 1 (Route B) toward quinazolidinones with various amino-acid side chains followed the protocol previously published by our group [16]. In brief, the synthesis began with a reaction between commercially available isatoic anhydride and the corresponding amino-acid ethyl/methyl ester in the presence of K_2_CO_3_ in ACN solution at 60 °C for 18 h to afford amides **15a**–**g** (55–93% after purification). The cyclocarbonylation reactions of compounds **15a**–**g** using 1,1-carbonyldiimidazole (CDI) in tetrahydrofuran (THF) solution at 85 °C for 18 h yielded the quinazolinediones **16a**–**g** in 55% to quantitative yield after CC. The esters **16a**–**g** were subsequently hydrolyzed using LiOH in a THF/water mixture to afford the carboxylic acids **17a**–**g** without any further purification (24% to quantitative yield). The corresponding acids **17a**–**g** were then reacted with the *m*-anisidine side-chain to form an amide bond using 1-[bis(dimethylamino)methylene]-1H-1,2,3-triazolo [4,5-b]pyridinium 3-oxide hexafluorophosphate (HATU) as a coupling agent in the presence of triethylamine (Et_3_N) in dimethylformamide (DMF) to yield the quinazolidinone final products **2** and **5**–**10** in 14–77% yields after purification (see Supplementary Materials for detailed experiment and compound characterization). It is worth noting that compound **8** was prepared as a racemate.

Compounds **2** and **4**–**10** were evaluated for their in vitro antimalarial activities against the blood-stage *P. falciparum* 3D7 strain. The results in Table 2 show that only the in-house synthesized compound **2** containing a valine linker possessed a promising IC_50_ (3D7) of 219 nM, whereas many analogues containing a less hydrophobic linker (compounds **4**–**6** and **10**) showed antimalarial activity of above 10 µM. Interestingly, some derivatives containing a slightly more hindered hydrophobic side chain (compounds **7** and **9**) showed a mild antimalarial activity (around 1–3 µM) against 3D7. Interestingly, compound **8** as a racemate displayed potent sub-micromolar activity against 3D7 but its activity was still worse than that of its original counterpart (compound **2**). Our group is currently working on another lead optimization program based on the structure of compound **8**. The results shown in Table 1 and Table 2 did not provide any correlations between good drug-likeness properties and potent antimalarial activities; therefore, the in silico prediction was not further employed in the next round of lead optimization. This result led to further lead optimization based on the structure of compound **2** to probe the antimalarial SARs around the *m*-anisidine side chain.

Another round of lead optimization focused on the SARs around the *m*-anisidine side chain of the valinyl quinazolinedione derivatives. It is well perceived that a methoxy group in a hit candidate could be replaced with a halogen or a nitrogen atom due to their similarity in size and their electronic properties [24]; for this reason, the substitution pattern on the aromatic side chain of quinazolinedione **2** was briefly investigated.

**Scheme 2:**
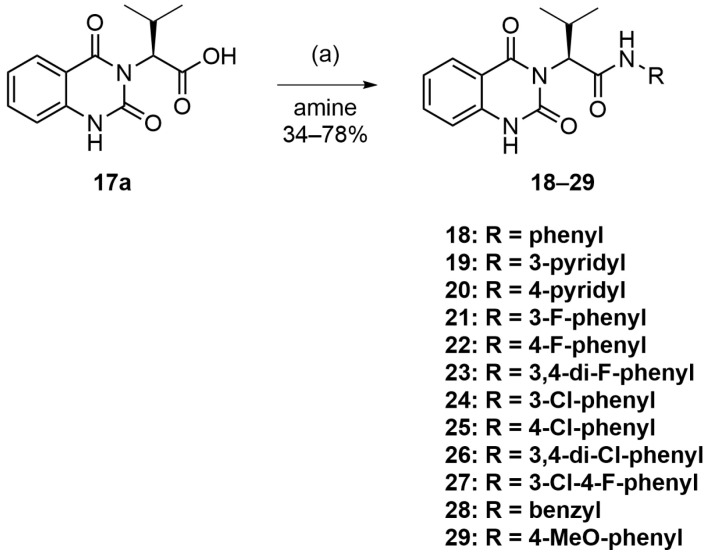
Reagents and conditions: (**a**) amines, HATU, Et_3_N, DMF, rt, 18 h.

The synthesis of compounds **18**–**29** is described in Scheme 2, based on the synthesis of parent compound **2**, as depicted previously in Scheme 1 (Route B). The synthesis was proven to be robust and compatible with various aromatic amine side chains. The resulting compounds **18**–**29** were obtained in 34–78% yield (see Supplementary Materials for compound characterization) and were then assayed for their antimalarial activity against blood-stage *P. falciparum* 3D7, as well as their cytotoxicity against MCF-7 cells.

As depicted in Table 3, the in vitro antimalarial data showed that excellent activity could be maintain by replacing the methoxy substituent in the meta position of the phenyl side chain with a fluorine or chlorine atom (compound **21** and **24**). A trend was observed in this series, whereby 3-fluoro and 3-chloro analogues provided optimal activity, and the addition of a small fluorine substituent at the 4-position did not dramatically affect the antimalarial activity (see comparisons of compound **1** and compound **21** for 3-fluoro, and compound **24** and **27** for 3-chloro). This 4-position blocking by a fluorine atom is a useful common strategy in a lead optimization to improve the metabolic stability of a lead compound. In contrast, removal of this methoxy group or replacing the *m*-anisidyl side chain with a benzyl or a *p*-anisidyl side chain (compound **18** and **28**–**29**, respectively) resulted in a dramatic loss in the antimalarial activity observed. Interestingly, derivatives with a halogen (F or Cl) at the 4-position of the phenyl possessed good antimalarial activity (compound **22** and **25**). Although the 3-chlorophenyl analogue (compound **24**) provided the best activity, we also noted that analogue **26**, which contained another chloro substituent at the 4-position, was a less potent antimalarial. Though it is widely accepted that fluorobenzene is a good bioisostere of pyridine due to their similarities in size and electron density, it was to our surprise that compounds **19** and **20** showed a decrease in antimalarial activity when compared to their fluorobenzene counterparts (compounds **21** and **22**, respectively) by up to an order of magnitude.

Compounds **18**–**29** were also assayed for their cytotoxicity against MCF-7 cells, and the results showed that only compounds **23** and **26** showed a slightly higher cytotoxicity against MCF-7 cells when compared with the parent compound. This suggests that the synthesized quinazolinediones derivatives may not be suitable as potent anti-MCF-7 agents.

In conclusion, a concise four-step synthesis of an array of valinyl quinazolinediones with potent antimalarial activity was successfully established with good overall yields, low cost of goods, and mild reaction conditions with the potential for scaling up. Although the chemical design was preliminarily guided by in silico predictions using SwissADME to predict any unwanted properties, any subtle changes to the hydrophobicity of the valine side chain dramatically affected the antimalarial activity. Most of the derivatives from this series showed no antimalarial activity at 10 µM. Only compound **2** possessed a potent IC_50_ of 219 nM. Further lead modification on the *m*-anisidine moiety of compound **2** led to the identification of more potent analogues **21** and **24** (**21**; IC_50_ = 36 ± 5 nM, **24**; IC_50_ = 22 ± 5 nM). Continuing lead optimization is underway to enhance the antimalarial activity of this series of compounds described in this work. The results from this work can encourage the selection of molecules in this class for additional in vitro DMPK, target identification, and in-depth hit-to-lead optimization campaigns in the near future.

## 3. Materials and Methods

### 3.1. Chemistry

All chemicals, reagents, and solvents were purchased from commercial suppliers (Sigma-Aldrich, Merck, or Tokyo Chemical Industry) and were used as such. NMR spectra were recorded on either a Bruker Avance AV400 (400/100 MHz for ^1^H/^13^C NMR) or Bruker Avance AV600 (600/150 MHz for ^1^H/^13^C NMR) spectrometer (Bruker, Billerica, MA, USA), and chemical shifts (δ, ppm) were downfield from the TMS reference. The chemical shifts are reported relative to residual the solvent signal in part per million (δ) (CD_3_OD: ^1^H: δ 3.31, ^13^C: δ 49.1; DMSO-d_6_: ^1^H: δ 2.50, ^13^C: δ 39.5; CDCl_3_: ^1^H: δ 7.26, ^13^C: δ 77.23). For the ^1^H-NMR spectra, data were assumed to be first-order with apparent singlets, doublets, triplets, quartets, and multiplets reported as s, d, t, q, and m, respectively. High-resolution mass spectral measurements were performed on either a Thermo Scientific Orbitrap Q Exactive Focus mass spectrometer (Thermo Fisher Scientific, Waltham, MA, USA) or a Bruker Daltonics maxis-UHR-TOF (Ultra High Resolution-TOF) (Bruker, Billerica, MA, USA). Thin-layer chromatography (TLC) was performed on a Merck aluminum sheet coated with silica gel 60 F_254_ (Merck, Darmstadt, Germany). UV lamps were used to visualize spots on the TLC sheet. The purification was performed on a Biotage^®^ Selekt Automated flash column chromatograph (Biotage, Uppsala, Sweden).

### 3.2. Synthesis

#### 3.2.1. General Procedure A (Scheme 3)

To a solution of ACN (75 mL) in a round-bottom flask, isatoic anhydride **14** (1 eq), amino acid ester (1 eq), and potassium carbonate (2.5 eq) were added. The reaction was allowed to stir, and then heated to 60 °C for 18 h. After that, the mixture was allowed to cool to room temperature, and then evaporated to remove solvent. The resulting residue was then stirred in a 0.4 M Na_2_CO_3_ solution for 1 h, and the mixture was extracted with CH_2_Cl_2_. The organic phase was collected, dried with anhydrous MgSO_4_, and evaporated to dryness by a rotary evaporator. Purification was performed using column chromatography (CC) over silica gel (10–30% ethyl acetate (EtOAc)/Hexanes) to yield compounds **15a**–**g**.

**Scheme 3:**
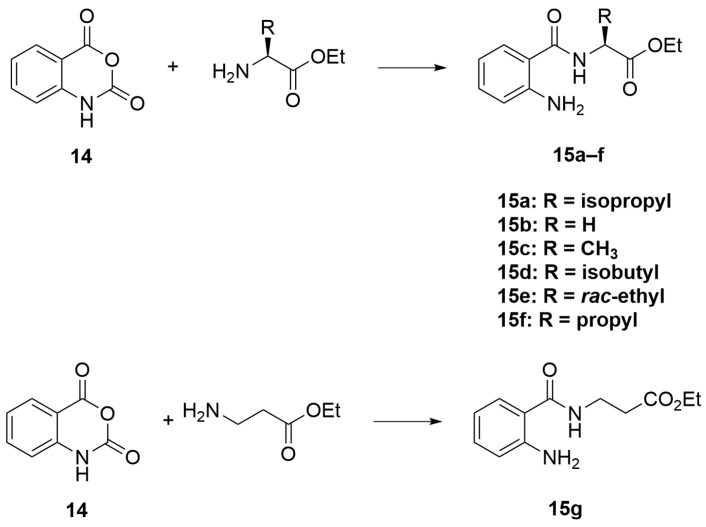
General procedure A.

#### 3.2.2. General Procedure B (Scheme 4)

To a solution of compounds **15a**–**g** (1 eq) in THF (40 mL), CDI (2 eq) was added. The reaction was allowed to stir for 18 h at 85 °C. When completed, the reaction was concentrated by a rotary evaporator. The resulting residue was then dissolved in EtOAc, washed with water, and dried over MgSO_4_. The organic portion was filtered and concentrated to give a crude product. Purification was performed using CC over silica gel (10–30% EtOAc/hexanes) to obtain the cyclized products **16a**–**16g**.

**Scheme 4:**
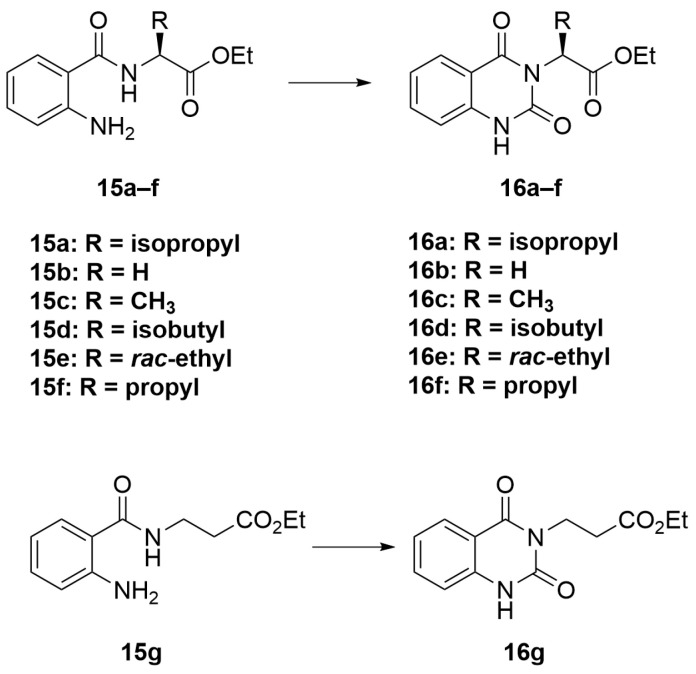
General procedure B.

#### 3.2.3. General Procedure C (Scheme 5)

A solution of LiOH (2.5 eq) in water (6 mL) was added to a solution of compounds **7**–**12** (1 eq) in THF (20 mL). The reaction mixture was heated and stirred at 95 °C for 18 h. After that, the mixture was allowed to cool down to room temperature and was concentrated under reduced pressure. The residue was dissolved in 10 mL of water and acidified with 1 M HCl. The white precipitate was collected and washed successively with methanol to afford the acid intermediates **17a**–**g** without further purification.

**Scheme 5:**
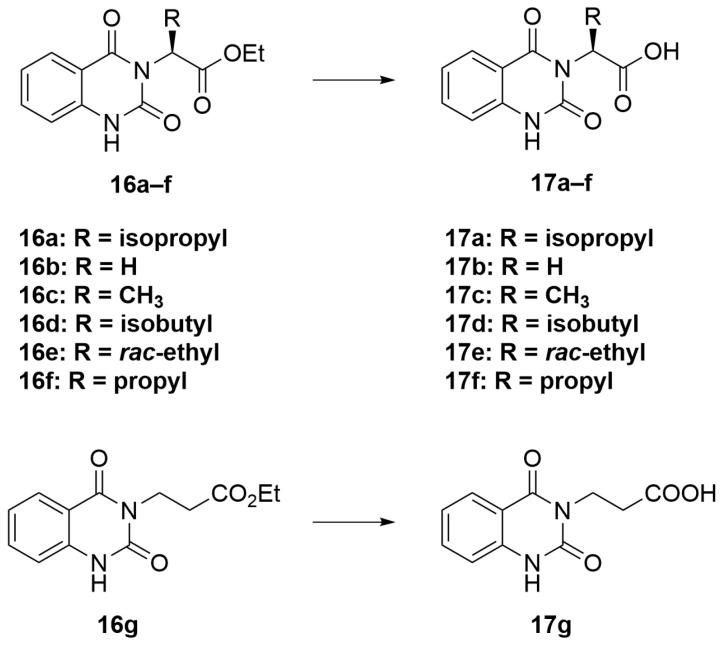
General procedure C.

#### 3.2.4. General Procedure D (Scheme 6)

To a solution of carboxylic acids **17a**–**17f** (1 eq) in DMF (4 mL), triethylamine (1 eq) and HATU (1 eq) were added. The mixture was left to stir for 1 h at room temperature. After that, amine (1.5 eq) was added, and the reaction mixture was left to stir at room temperature for 18 h. After the reaction was completed, the solvent was removed under reduced pressure. The residue was dissolved in EtOAc; the organic solution was extracted with 0.4 M Na_2_CO_3_ solution and washed with water. The organic layer was collected and dried over MgSO_4_; the solvent was evaporated under reduced pressure. Purification was performed using CC over silica gel or an automated flash column chromatograph (Biotage^®^) (10–50% EtOAc/hexanes), or recrystallization was performed with EtOAc to afford the desired quinazolinedione products **2**, **5**–**10**, and **18**–**29**.

**Scheme 6:**
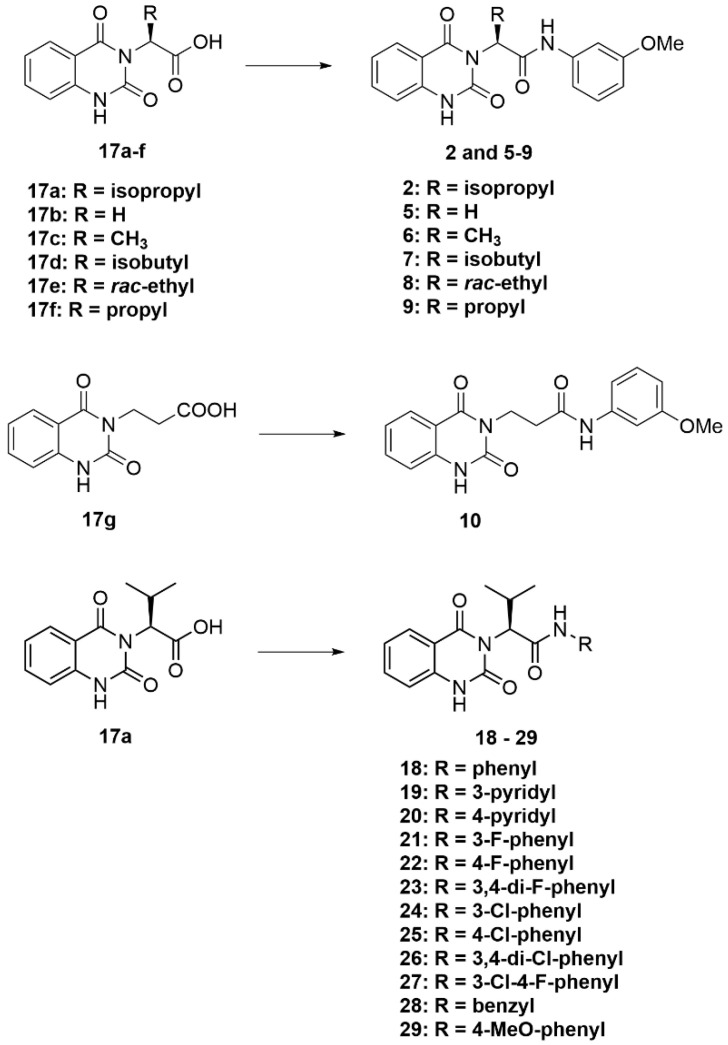
General procedure D.

### 3.3. Antimalarial Assay against P. falciparum 3D7

*Plasmodium falciparum* strain 3D7 was cultured in complete medium (RPMI-1640 supplemented with 10% Albumax II) using O Rh^+^ red blood cells in a microaerobic environment (5% CO_2_, 5% O_2_, 90% N_2_). IC_50_ assay plates were prepared using fourfold serially diluted test compounds in complete medium to a final volume of 50 µL. Then, 50 µL of parasite inoculum at 2% parasitemia ring stage and 1% hematocrit was added to each well and incubated for 48 h in a microaerobic environment. The assay was terminated by freezing at −20 °C before growth measurement. Parasite growth was measured adding 100 µL of lysis buffer supplemented with 1× DNA fluorescent dye (UltraPower, Gellex, Tokyo, Japan), and the fluorescent signal was measured at 495/530 nm. The IC_50_ value was calculated by GraphPad Prism 9.0 software (La Jolla, California, USA) using the dose–response (four-parameter) function. Artemisinin at 1 µM and complete medium were used as positive and negative controls, respectively.

### 3.4. Antiproliferative Assay against MCF-7

Human breast cancer cells (MCF-7) purchased from ATCC were seeded at 2 × 10^3^ cells/well on a 96-well black flat-bottom plate and were cultured in high-glucose DMEM (Dulbecco’s modified Eagle medium) supplemented with 10% fetal bovine serum and 1% penicillin/streptomycin. The culture was incubated at 37 °C, 5% CO_2_ for 24 h. After the incubation period, the test compounds were added into the cell plate at serially diluted concentrations (20, 2, 0.2, 0.02, 0.002, and 0.0002 μM), and the culture was incubated for 72 h at 37 °C, 5% CO_2_. After the 72 h incubation, the cultured media containing compounds were removed, and the serum-free media containing MTT were added to the same well with additional incubation for 3 h at 37 °C, 5% CO_2_. After 3 h incubation, the serum-free media containing MTT were removed, and DMSO was added into the same well; the resulting solution was measured for its absorbance at 570 nm using a Multimode Microplate Reader (ENVISION) (PerkinElmer, USA). The IC_50_ value was calculated using GraphPad. Doxorubicin at 10 µM was used as a positive control in this assay.

## Figures and Tables

**Table 1:** SwissADME in silico calculation of TCMDC-125133 and other derivatives containing various side chains. Red font denotes unsuitable properties for a drug candidate, orange font denotes moderate suitability, and green font denotes a favorable profile.

Structure	Consensus Log P	LogS (ESOL)	Pharmacokinetics					Lead-Likeness	Synthetic Tractability
			GI Absorption	BBB Permeant	Pgp Substrate	CYP1A2 Inhibitor	CYP2C9 Inhibitor		
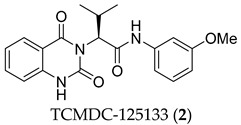	2.71	−3.97	High	No	No	No	Yes	1 violation: MW > 350	3.29
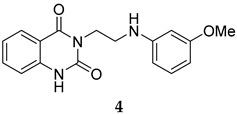	2.39	−3.83	High	No	No	Yes	Yes	Yes	2.54
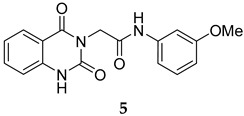	1.76	−2.97	High	No	No	No	No	Yes	2.55
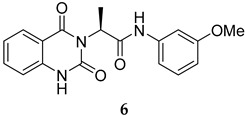	2.08	−3.28	High	No	No	No	Yes	Yes	3.07
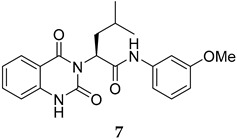	2.91	−4.19	High	No	No	No	Yes	1 violation: MW > 350	3.4
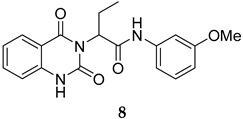	2.39	−3.62	High	No	No	No	Yes	1 violation: MW > 350	3.18
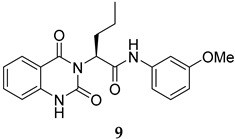	2.74	−3.85	High	No	No	Yes	Yes	1 violation: MW > 350	3.29
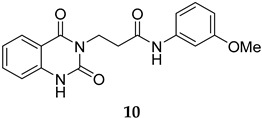	1.99	−2.90	High	No	No	No	No	Yes	2.55

**Table 2:** In vitro antimalarial activities against *P. falciparum* 3D7 of quinazolinedione derivatives with various amino-acid linkers.

Compound	Structure	IC_50_ (µM) 3D7 ± SD
**2**	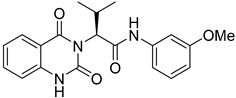	0.219 ± 0.024
**4**	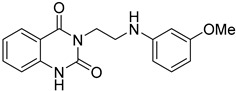	>10
**5**	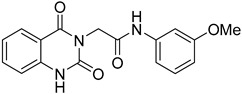	>10
**6**	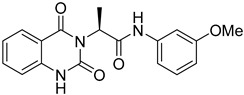	>10
**7**	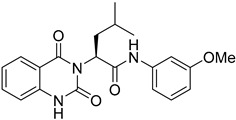	3.25 ± 0.21
**8**	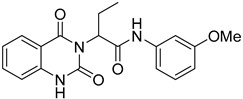	0.586 ± 0.032
**9**	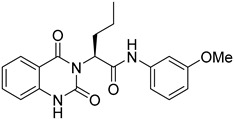	1.11 ± 0.10
**10**	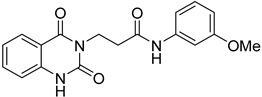	>10

**Table 3:** In vitro antimalarial activities against *P. falciparum* 3D7 and cytotoxicity against MCF-7 cells of quinazolinedione derivatives with various amine side chains.

Compound	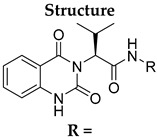	IC_50_ (µM) 3D7 ± SD	IC_50_ (µM) MCF-7
**2**	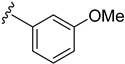	0.219 ± 0.024	17.50
**18**	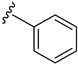	0.607 ± 0.030	>20
**19**	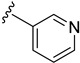	2.886 ± 0.401	>20
**20**	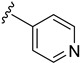	1.407 ± 0.115	>20
**21**	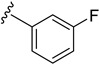	0.036 ± 0.005	>20
**22**	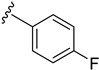	0.360 ± 0.034	>20
**23**	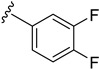	0.038 ± 0.009	9.74
**24**	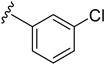	0.022 ± 0.005	>20
**25**	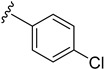	0.711 ± 0.038	10.52
**26**	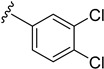	0.406 ± 0.034	>20
**27**	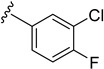	0.059 ± 0.012	>20
**28**	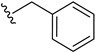	>10	>20
**29**	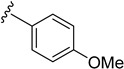	>10	>20

## Data Availability

The data presented in this study are available in the Supplementary Materials and on request from the corresponding author.

## Supplementary Materials

The following supporting information can be downloaded at https://www.mdpi.com/article/10.3390/molecules28072999/s1 including ^1^H-NMR, ^13^C-NMR, and HRMS data of compounds **2**, **4**–**10**, **12**, and **14**–**29**.
