# Supplementary material for: Rational Design and Lead Optimisation of Potent Antimalarial Quinazolinediones and Their Cytotoxicity against MCF-7
Source: Molecules. 2023 Mar 28;28(7):2999. doi: 10.3390/molecules28072999 (PMC10096129; doi:10.3390/molecules28072999)
Supplement: Supplementary file 1 [file molecules-28-02999-s001.zip › molecules-2283025-supplementary.pdf]

Rational Design and Lead Optimisation of Potent Antimalarial Quinazolinediones and Their Cytotoxicity against MCF-7

Sitthivut Charoensutthivarakul [1, 2, 3] *, Duangporn Lohawittayanan [1], Phongthon Kanjanasirirat [2], Kedchin Jearawuttanakul [2], Sawinee Seemakhan [2], Napason Chabang [1], Patrick Schlaeppi [1], Varisa Tantivess [1], Tanapol Limboonreung and Matthew Phanchana [5]

[1]Innovative Molecular Discovery Laboratory (iMoD), School of Bioinnovation and Bio-based Product Intelligence, Faculty of Science, Mahidol University, Bangkok, 10400, Thailand

[2]Excellent Center for Drug Discovery (ECDD), Faculty of Science, Mahidol University, Bangkok, 10400, Thailand

[3]Center for Neuroscience, Faculty of Science, Mahidol University, Bangkok, 10400, Thailand

[4]School of Dentistry, King Mongkut's Institute of Technology Ladkrabang, Chalongkrung Road, Ladkrabang, Bangkok 10520, Thailand

[5]Department of Molecular Tropical Medicine and Genetics, Faculty of Tropical Medicine, Mahidol University, Bangkok, 10400, Thailand

*Correspondence: sitthivut.cha@mahidol.ac.th

Preparation of compound **12**

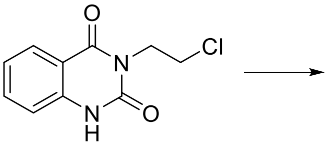

11 **12**

To an acetonitrile solution (32 mL) of commercially available 3-(2-chloroethyl)-2,4-quinazolinedione (**11**) (2.00 g, 8.90 mmol, 1 eq), potassium carbonate (2.46 g, 17.80 mmol, 2 eq) and potassium iodide (0.15 g, 8.90 mmol, 1 eq) was added in round bottom flask. The reaction was allowed to stir and heated to 80 °C for 18 hours. After that the mixture was allowed to cool to room temperature and evaporated to remove solvent. The crude was dissolved with CH_2Cl_2 (30 mL) and was extracted with water (30 mL x 3). The organic phase was collected, dried over with anhydrous $MgSO_4$ and evaporated by rotary evaporator to yield compound **12** as white solid (1.62g, 97%).

^{1}H-NMR (400 MHz, $CDCl_3$) δ 8.14 (dd, *J* = 8.0, 1.2 Hz, 1H), 7.65 (ddd, *J* = 8.5, 7.2, 1.6 Hz, 1H), 7.53 – 7.45 (m, 1H), 7.35 – 7.28 (m, 1H), 4.74 (dd, *J* = 9.8, 6.7 Hz, 2H), 4.36 (t, *J* = 8.2 Hz, 2H). ^{13}C-NMR (101 MHz, $CDCl_3$) δ 160.99, 155.60, 149.08, 134.94, 126.70, 126.32, 124.89, 118.51, 65.94, 42.35. ESI-HRMS (*m/z*): found 186.0662 $[M+H]^+$ (calcd for $C_{10}H_9N_2O_2^+$ 186.0659).

Preparation of compound **4**

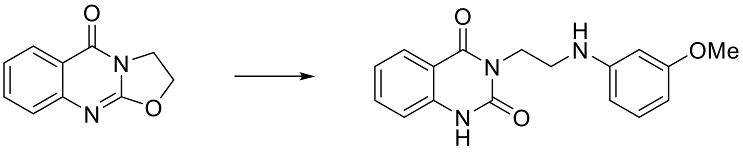

12 **4**

To a solution of *m*-anisidine (0.168 mL, 1.5 mmol, 1.5 eq) in acetronitrile (4 mL) was treated in 25 mL round bottom flask. The reaction was allowed to stir and gently heated to increase the solubility of the reaction mixture. Compound **12** (0.189 g, 1.0 mmol, 1.0 eq) was then added into reaction mixture flask and the reaction was allowed to heat at 120 °C for 48 hours. After that the mixture was allowed to cool to room temperature and the solvent was evaporated using a rotatory evaporator. Purification was performed by an automatic column chromatography (Biotage) eluting with 0-100% CH_2Cl_2/hexanes following by 10-50% EtOAc/CH_2Cl_2 to obtain compound **4** as off-white solid (0.049 g, 16% yield).

^{1}H-NMR (600 MHz, $CDCl_3$) δ 9.94 (s, 1H, NH in ring), 8.14 (dd, *J* = 7.9, 1.3 Hz, 1H), 7.62 (td, *J* = 7.8, 1.6 Hz, 1H), 7.25 – 7.23 (m, 1H), 7.10 (d, *J* = 8.1 Hz, 1H), 7.04 (t, *J* = 8.0 Hz, 1H), 6.28 (dd, *J* = 8.1, 2.2 Hz, 1H), 6.25 (t, *J* = 2.2 Hz, 1H), 6.23 (dd, *J* = 8.0, 2.3 Hz, 1H), 4.39 (t, *J* = 6.3 Hz, 2H), 4.29 (s, 1H, NH amine), 3.74 (s, 3H), 3.51 (t, *J* = 6.3 Hz, 2H). ^{13}C-NMR (151 MHz, $CDCl_3$) δ 162.98, 161.01, 152.31, 149.44, 138.60, 135.40, 130.12, 128.60, 123.73, 115.13, 114.64,

105.79, 102.89, 98.53, 58.63, 55.23, 42.68. ESI-HRMS (*m/z*): found 312.1344 $[M+H]^+$ (calcd for $C_{17}H_{18}N_3O_3^+$ 312.1343).

General procedure A; (Preparation of compound **15a**-**g**)

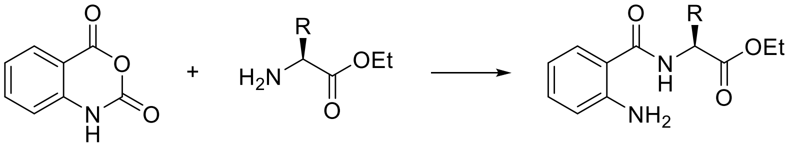

14 **15a-f**

15a: R = isopropyl
15b: R = H
15c: R = CH_3
15d: R = isobutyl
15e: R = *rac*-ethyl
15f: R = propyl

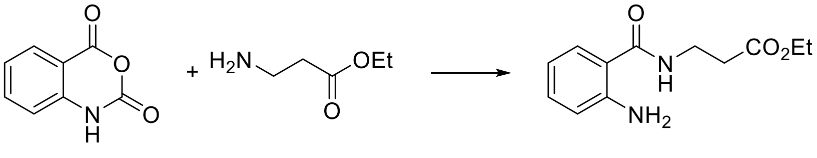

14 **15g**

To a solution of acetonitrile (75 mL) in round bottom flask, isatoic anhydride **14** (1 eq), amino acid ester (1 eq), and potassium carbonate (2.5 eq) were added. The reaction was allowed to stir and heated to 60 °C for 18 hours. After that the mixture was allowed to cool to room temperature and evaporated to remove solvent. The resulting residue was then stirred in a 0.4 M Na_2CO_3 solution for an hour and the mixture was extracted with CH_2Cl_2. The organic phase was collected, dried with anhydrous $MgSO_4$, and evaporated to dryness by a rotary evaporator. Purification was performed using column chromatography (CC) over silica gel (10-30 % EtOAc/hexanes) to yield compound **15a-g**.

Preparation of compound **15a**

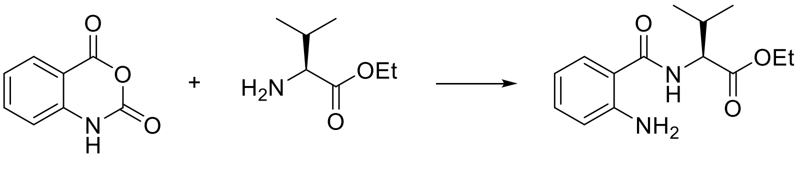

14 **15a**

ethyl (2-aminobenzoyl)-L-valinate (**15a**): yellow oil, 1.39 g, 55% yield. FTIR (cm^{-1}): 3457.7 (NH-C=O), 3380.8 and 3353.5 (NH_2), 2967.7, 2925.7 and 2873.8 (=C-H aromatic), 1735.4 (O-C=O), and 1638.2 (O=C-NH). ^{1}H-NMR (400 MHz, $CDCl_3$): δ 7.40 (d, *J* = 7.80 Hz, 1H), 7.17 (td, *J* = 5.44, 7.68 Hz, 1H), 6.65-6.61 (m, 2H), 4.57 (dd, *J* = 4.96, 8.54, 1H), 4.26-4.13 (m, 2H), 2.27-2.19 (m, 1H), 1.27 (t, *J* = 7.16 Hz, 3H), 0.98 (d, *J* = 6.88 Hz, 3H), 0.95 (d, *J* = 6.88 Hz,

3H). ^{13}C-NMR (100 MHz, $CDCl_3$); δ 172.17, 168.99, 148.66, 132.39, 127.35, 117.18, 116.51, 115.62, 61.24, 57.01, 31.40, 18.91, 17.88, 14.13. ESI-HRMS (*m/z*): found 265.1541 [M+H]$^+$ (calcd for $C_{14}H_{21}N_2O_3^+$ 265.1547).

Preparation of compound **15b**

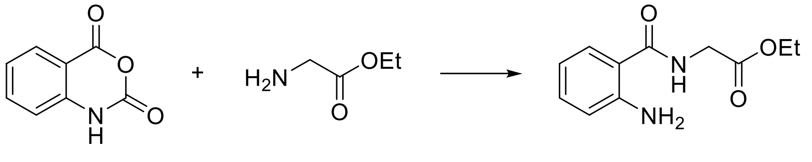

14 **15b**

ethyl (2-aminobenzoyl)glycinate (**15b**): white solid, 1.45 g, 64% yield. ^{1}H-NMR (400 MHz, $CDCl_3$) δ 7.38 (dd, *J* = 7.9, 1.1 Hz, 1H), 7.17 (td, *J* = 7.7, 1.4 Hz, 1H), 6.79 (s, 1H, NH), 6.64 (d, *J* = 8.3 Hz, 1H), 6.61 (t, *J* = 7.6 Hz, 1H), 4.20 (q, *J* = 7.1 Hz, 2H), 4.12 (d, *J* = 5.2 Hz, 2H), 1.26 (t, *J* = 7.1 Hz, 3H). ^{13}C-NMR (101 MHz, $CDCl_3$) δ 170.32, 169.42, 148.78, 132.61, 127.60, 117.31, 116.66, 115.23, 61.59, 41.59, 14.14. ESI-HRMS (*m/z*): found 223.1075 [M+H]$^+$ (calcd for $C_{11}H_{15}N_2O_3^+$ 223.1077).

Preparation of compound **15c**

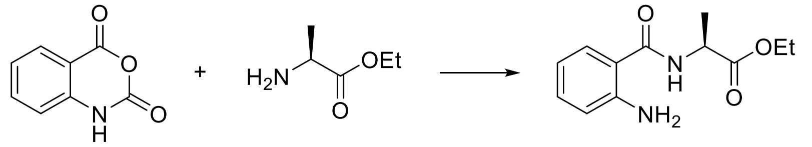

14 **15c**

ethyl (2-aminobenzoyl)-L-alaninate (**15c**): pale yellow solid, 3.06 g, 86% yield. ^{1}H-NMR (400 MHz, $CDCl_3$) δ 7.40 (dd, *J* = 7.8, 1.0 Hz, 1H), 7.24 – 7.15 (m, 1H), 6.68 – 6.64 (m, 2H), 4.72 (p, *J* = 7.2 Hz, 1H), 4.23 (q, *J* = 7.1 Hz, 2H), 1.50 (d, *J* = 7.1 Hz, 3H), 1.30 (t, *J* = 7.1 Hz, 3H). ^{13}C-NMR (101 MHz, $CDCl_3$) δ 173.45, 168.79, 148.83, 132.67, 127.57, 117.47, 116.83, 115.57, 61.71, 48.38, 18.77, 14.27. ESI-HRMS (*m/z*): found 237.1232 [M+H]$^+$ (calcd for $C_{12}H_{17}N_2O_3^+$ 237.1234).

Preparation of compound **15d**

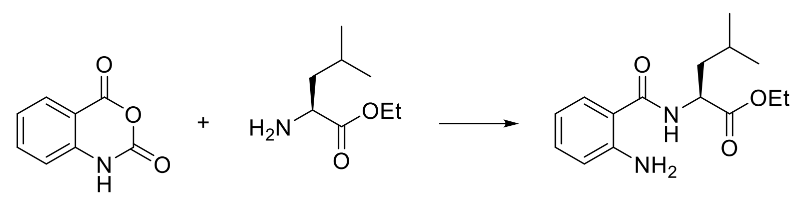

14 **15d**

ethyl (2-aminobenzoyl)-L-leucinate (**15d**): yellow oil, 2.72 g, 65% yield. ^{1}H-NMR (400 MHz, $CDCl_3$) δ 7.39 (dd, *J* = 8.2, 1.3 Hz, 1H), 7.21 (td, *J* = 8.1, 1.4 Hz, 1H), 6.69 – 6.64 (m, 2H), 6.43 (d, *J* = 7.9 Hz, 1H, NH), 5.48 (s, 2H, NH_2), 4.78 (td, *J* = 8.5, 5.1 Hz, 1H), 4.22 (q, *J* = 7.1 Hz, 2H), 1.78 – 1.70 (m, 2H), 1.66 – 1.61 (m, 1H), 1.30 (t, *J* = 7.1 Hz, 3H), 0.98 (t, *J* = 5.9 Hz, 6H). ^{13}C-NMR (151 MHz, $CDCl_3$) δ 173.45, 169.03, 148.86, 132.58, 127.53, 117.35, 116.68, 115.61, 61.48, 50.95, 41.84, 25.06, 22.92, 22.16, 14.24. ESI-HRMS (*m/z*): found 279.1709 $[M+H]^+$ (calcd for $C_{15}H_{23}N_2O_3^+$ 279.1703).

Preparation of compound **15e**

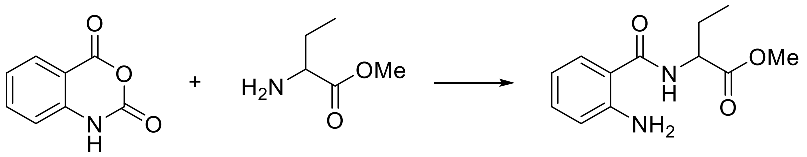

14 **15e**

methyl 2-(2-aminobenzamido)butanoate (**15e**): dark yellow oil, 2.81 g, 79% yield. ^{1}H-NMR (400 MHz, $CDCl_3$) δ 7.41 (d, *J* = 7.1 Hz, 1H), 7.21 (td, *J* = 8.1, 1.5 Hz, 1H), 6.68 (d, *J* = 7.7 Hz, 1H), 6.65 (dd, *J* = 7.4, 1.0 Hz, 1H), 6.61 (d, *J* = 6.8 Hz, 1H, NH), 4.76 – 4.70 (m, 1H), 3.78 (s, 3H), 2.03 – 1.93 (m, 1H), 1.88 – 1.77 (m, 1H), 0.97 (t, *J* = 7.5 Hz, 3H). ^{13}C-NMR (101 MHz, $CDCl_3$) δ 173.27, 168.98, 148.79, 132.72, 127.55, 117.51, 116.88, 115.61, 53.45, 52.54, 25.87, 9.72. ESI-HRMS (*m/z*): found 237.1235 $[M+H]^+$ (calcd for $C_{12}H_{17}N_2O_3^+$ 237.1234).

Preparation of compound **15f**

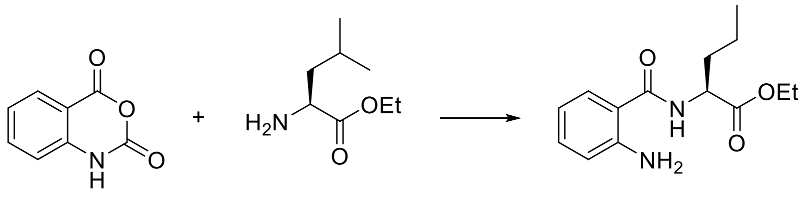

14 **15f**

ethyl (S)-2-(2-aminobenzamido)pentanoate (**15f**): off-white solid, 3.03 g, 76% yield. ^{1}H-NMR (400 MHz, $CDCl_3$) δ 7.40 (d, *J* = 7.2 Hz, 1H), 7.21 (td, *J* = 8.0, 1.4 Hz, 1H), 6.67 (d, *J* = 7.7 Hz, 2H), 6.57 (d, *J* = 7.4 Hz, 1H, NH), 5.49 (s, 2H, NH_2), 4.75 (td, *J* = 7.5, 5.5 Hz, 1H), 4.27 – 4.18 (m, 2H), 1.95 – 1.86 (m, 1H), 1.79 – 1.69 (m, 1H), 1.48 – 1.35 (m, 2H), 1.30 (t, *J* = 7.1 Hz, 3H), 0.95 (t, *J* = 7.3 Hz, 3H). ^{13}C-NMR (101 MHz, $CDCl_3$) δ 173.02, 168.96, 148.91, 132.64, 127.55, 117.40, 116.76, 115.65, 61.57, 52.26, 34.90, 18.77, 14.32, 13.88. ESI-HRMS (*m/z*): found 265.1546 $[M+H]^+$ (calcd for $C_{14}H_{21}N_2O_3^+$ 265.1547).

Preparation of compound **15g**

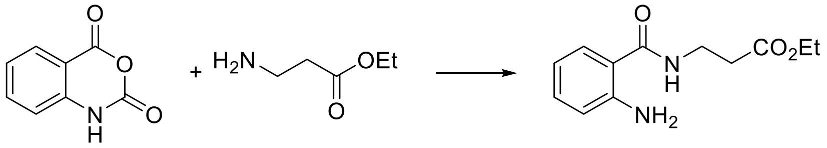

14 **15g**

ethyl 3-(2-aminobenzamido)propanoate (**15g**): white solid, 6.59 g, 93% yield. ^{1}H-NMR (400 MHz, $CDCl_3$) δ 7.22 (dd, *J* = 7.9, 1.1 Hz, 1H), 7.08 (td, *J* = 7.7, 1.4 Hz, 1H), 6.83 (s, 1H, NH), 6.57 (d, *J* = 8.2 Hz, 1H), 6.52 (td, *J* = 7.5, 0.9 Hz, 1H), 5.44 (s, 2H, NH_2), 4.06 (q, *J* = 7.1 Hz, 2H), 3.55 (q, *J* = 6.1 Hz, 2H), 2.52 (t, *J* = 6.1 Hz, 2H), 1.16 (t, *J* = 7.1 Hz, 3H). ^{13}C-NMR (101 MHz, $CDCl_3$) δ 172.77, 169.29, 148.72, 132.24, 127.30, 117.20, 116.51, 115.81, 60.74, 35.00, 34.01, 14.12. ESI-HRMS (*m/z*): found 237.1230 $[M+H]^+$ (calcd for $C_{12}H_{17}N_2O_3^+$ 237.1234).

General procedure B; (Preparation of compound **16a**-**g**)

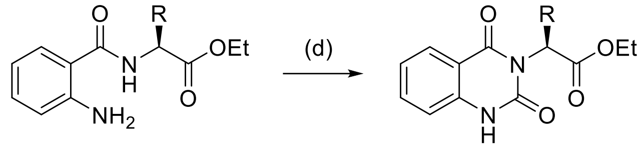

15a-f **16a-f**

15a: R = isopropyl
15b: R = H
15c: R = CH_3
15d: R = isobutyl
15e: R = *rac*-ethyl
15f: R = propyl

16a: R = isopropyl
16b: R = H
16c: R = CH_3
16d: R = isobutyl
16e: R = *rac*-ethyl
16f: R = propyl

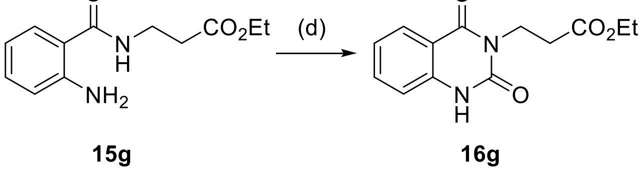

15g **16g**

To a solution of compound **15a-g** (1 eq) in THF (40 mL), 1,1'-carbonyldiimidazole (CDI) (2 eq) was added. The reaction was allowed to stir 18 hours at 85 °C. When completed, the reaction was concentrated by a rotary evaporator. The resulting residue was then dissolved in EtOAc, washed with water, and dried over $MgSO_4$. The organic portion was filtered and concentrated to give a crude product. Purification was performed using CC over silica gel (10-30% EtOAc/hexanes) to obtain the intermediate cyclized products **16a-16g**.

Preparation of compound **16a**

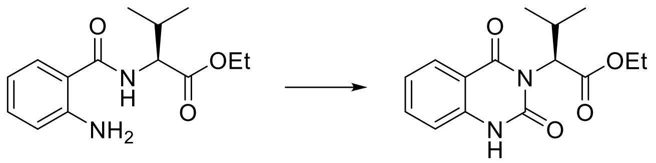

15a **16a**

ethyl (S)-2-(2,4-dioxo-1,4-dihydroquinazolin-3(2H)-yl)-3-methylbutanoate (**16a**): yellow oil, 1.49 g, 97% yield. FTIR (cm^{-1}): 3253.5 (NH-C=O), 2965.5, 2928.6 and 2873.6 (=C-H aromatic), 1746.6 (O-C=O), 1716.9 (O=C-N-R), and 1656.4 (O=C-NH). ^{1}H-NMR (400 MHz, MeOD-d_4): δ = 8.02 (dd, *J* = 1.2, 8.0 Hz, 1H), 7.67 (td, *J* = 1.4, 7.8 Hz, 1H), 7.25 (t, *J* = 7.9 Hz, 1H), 7.19 (d, *J* = 8.2 Hz, 1H), 5.12 (d, *J* = 9.3 Hz, 1H), 4.21-4.07 (m, 2H), 2.77–2.68 (m, 1H), 1.67 (d, *J* = 6.5 Hz, 3H), 1.16 (t, *J* = 7.1 Hz, 3H), 0.76 (d, *J* = 6.9 Hz, 3H). ^{13}C-NMR (100 MHz, MeOD-d_4); δ = 171.22, 164.09, 152.11, 140.84, 136.79, 129.16, 124.43, 116.33, 114.95, 62.25, 60.12, 28.69, 22.51, 19.26, 14.45. ESI-HRMS (*m/z*): found 291.1335 $[M + H]^+$ (calcd for $C_{15}H_{18}N_2O_4^+$ 291.1339).

Preparation of compound **16b**

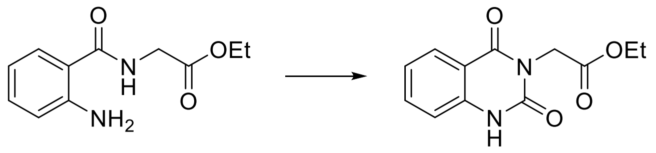

15b **16b**

ethyl 2-(2,4-dioxo-1,4-dihydroquinazolin-3(2H)-yl)acetate (**16b**): white solid, 1.25 g, 77% yield. ^{1}H-NMR (400 MHz, $CDCl_3$) δ 7.94 (d, *J* = 8.0 Hz, 1H), 7.52 – 7.46 (m, 1H), 7.09 (t, *J* = 7.6 Hz, 1H), 7.02 (d, *J* = 8.2 Hz, 1H), 4.65 (s, 1H), 4.10 (d, *J* = 7.1 Hz, 2H), 3.96 (s, 2H), 1.16 (t, *J* = 7.1 Hz, 3H). ^{13}C-NMR (101 MHz, $CDCl_3$) δ 168.31, 164.48, 162.55, 139.16, 135.35, 128.06, 123.20, 115.13, 113.88, 61.67, 41.66, 13.83. ESI-HRMS (*m/z*): found 249.0869 $[M+H]^+$ (calcd for $C_{12}H_{13}N_2O_4^+$ 249.0870).

Preparation of compound **16c**

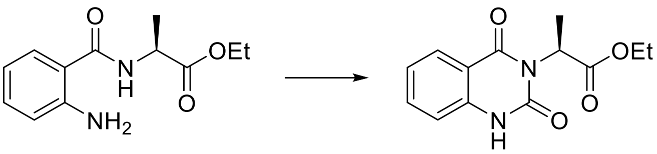

15c **16c**

(S)-ethyl-2-(2,4-dioxo-1,4-dihydroquinazolin-3(2H)-yl)propanoate (**16c**): pale yellow solid, 0.39 g, 70% yield. ^{1}H-NMR (400 MHz, $CDCl_3$) δ 10.82 (s, 1H, NH), 8.16 (dd, *J* = 8.0, 1.0 Hz,

1H), 7.66 (td, *J* = 7.7, 1.4 Hz, 1H), 7.29 (d, *J* = 6.2 Hz, 1H), 7.16 (d, *J* = 8.1 Hz, 1H), 5.68 (q, *J* = 7.0 Hz, 1H), 4.31 – 4.19 (m, 2H), 1.72 (d, *J* = 7.0 Hz, 3H), 1.26 (t, *J* = 7.1 Hz, 3H). ^{13}C-NMR (101 MHz, $CDCl_3$) δ 170.32, 161.81, 151.82, 138.76, 135.48, 128.64, 123.76, 115.36, 114.59, 61.60, 49.85, 14.67, 14.27. ESI-HRMS (*m/z*): found 263.1025 $[M+H]^+$ (calcd for $C_{13}H_{15}N_2O_4^+$ 263.1026).

Preparation of compound **16d**

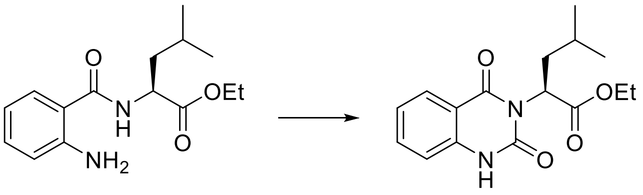

15d **16d**

(S)-ethyl-2-(2,4-dioxo-1,4-dihydroquinazolin-3(2H)-yl)-4-methylpentanoate (**16d**): white solid, 2.06 g, 59% yield. ^{1}H-NMR (400 MHz, $CDCl_3$) δ 10.14 (s, 1H, NH), 8.13 (d, *J* = 7.7 Hz, 1H), 7.63 (td, *J* = 7.7, 1.3 Hz, 1H), 7.25 (t, *J* = 7.6 Hz, 1H), 7.08 (d, *J* = 8.1 Hz, 1H), 5.64 (m, 1H), 4.24 – 4.16 (m, 2H), 2.20 – 2.10 (m, 2H), 1.59 – 1.50 (m, 1H), 1.21 (t, *J* = 7.1 Hz, 3H), 1.02 (d, *J* = 6.5 Hz, 3H), 0.94 (d, *J* = 6.6 Hz, 3H). ^{13}C-NMR (151 MHz, $CDCl_3$) δ 170.33, 162.17, 152.14, 138.82, 135.47, 128.79, 123.74, 115.27, 114.56, 61.59, 52.78, 37.94, 25.42, 23.34, 22.13, 14.28. ESI-HRMS (*m/z*): found 305.1490 $[M+H]^+$ (calcd for $C_{16}H_{21}N_2O_4^+$ 305.1496).

Preparation of compound **16e**

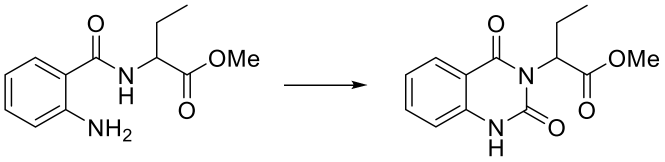

15e **16e**

methyl 2-(2,4-dioxo-1,4-dihydroquinazolin-3(2H)-yl)butanoate (**16e**): yellow oil, 3.54 g, 79% yield. ^{1}H-NMR (400 MHz, $CDCl_3$) δ 10.85 (s, 1H, NH), 8.13 (d, *J* = 7.9 Hz, 1H), 7.65 (td, *J* = 7.7, 1.0 Hz, 1H), 7.26 (t, *J* = 7.6 Hz, 1H), 7.15 (d, *J* = 8.1 Hz, 1H), 5.57 (dd, *J* = 9.8, 5.2 Hz, 1H), 3.75 (s, 3H), 2.48 – 2.33 (m, 1H), 2.30 – 2.17 (m, 1H), 0.99 (t, *J* = 7.5 Hz, 3H). ^{13}C-NMR (101 MHz, $CDCl_3$) δ 170.56, 162.22, 152.18, 138.85, 135.55, 128.75, 123.78, 115.38, 114.43, 55.28, 52.55, 22.03, 10.90. ESI-HRMS (*m/z*): found 263.1030 $[M+H]^+$ (calcd for $C_{13}H_{15}N_2O_4^+$ 263.1026).

Preparation of compound **16f**

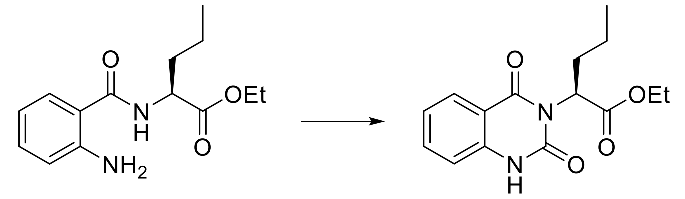

15f **16f**

(S)-ethyl-2-(2,4-dioxo-1,4-dihydroquinazolin-3(2H)-yl)pentanoate (**16f**): yellow oil, 3.75 g, quantitative yield. ^{1}H-NMR (400 MHz, $CDCl_3$) δ 8.11 (dd, *J* = 8.0, 1.1 Hz, 1H), 7.75 (s, 1H, NH), 7.61 (td, *J* = 7.7, 1.4 Hz, 1H), 7.23 (td, *J* = 7.6, 0.7 Hz, 1H), 7.14 (d, *J* = 8.1 Hz, 1H), 5.59 (dd, *J* = 9.7, 5.0 Hz, 1H), 4.24 – 4.15 (m, 2H), 2.34 – 2.23 (m, 1H), 2.23 – 2.12 (m, 1H), 1.44 – 1.37 (m, 1H), 1.36 – 1.28 (m, 1H), 1.21 (t, *J* = 7.1 Hz, 3H), 0.94 (t, *J* = 7.3 Hz, 3H). ^{13}C-NMR (101 MHz, $CDCl_3$) δ 170.25, 162.30, 151.90, 139.04, 135.41, 128.69, 123.60, 121.92, 115.31, 114.44, 61.53, 53.97, 30.84, 19.73, 14.25. ESI-HRMS (*m/z*): found 291.1343 $[M+H]^+$ (calcd for $C_{15}H_{19}N_2O_4^+$ 291.1339).

Preparation of compound **16g**

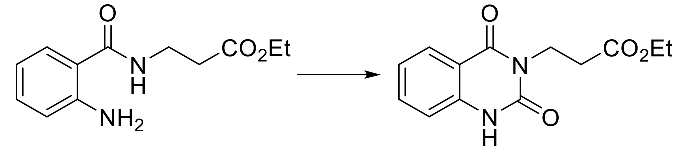

15g **16g**

ethyl 3-(2,4-dioxo-1,4-dihydroquinazolin-3(2H)-yl)propanoate (**16g**): white solid, 5.43 g, 78% yield. ^{1}H-NMR (400 MHz, $CDCl_3$) δ 10.87 (s, 1H, NH), 8.10 (d, *J* = 7.8 Hz, 1H), 7.60 (td, *J* = 7.7, 1.3 Hz, 1H), 7.21 (t, *J* = 7.6 Hz, 1H), 7.16 (d, *J* = 8.1 Hz, 1H), 4.40 (t, *J* = 7.5 Hz, 2H), 4.14 (q, *J* = 7.1 Hz, 2H), 2.75 (t, *J* = 7.4 Hz, 2H), 1.23 (t, *J* = 7.1 Hz, 3H). ^{13}C-NMR (101 MHz, $CDCl_3$) δ 171.46, 162.31, 152.25, 138.75, 135.28, 128.37, 123.59, 115.39, 114.54, 60.89, 36.89, 32.68, 14.22. ESI-HRMS (*m/z*): found 263.1023 $[M+H]^+$ (calcd for $C_{13}H_{15}N_2O_4^+$ 263.1026).

General procedure C; (Preparation of compound **17a**-**g**)

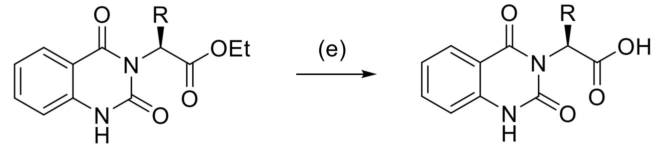

16a-f **17a-f**

16a: R = isopropyl
16b: R = H
16c: R = CH_3
16d: R = isobutyl
16e: R = *rac*-ethyl
16f: R = propyl

17a: R = isopropyl
17b: R = H
17c: R = CH_3
17d: R = isobutyl
17e: R = *rac*-ethyl
17f: R = propyl

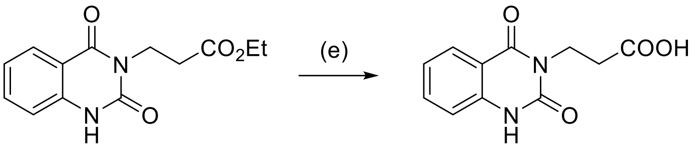

16g **17g**

A solution of LiOH (2.5 eq) in water (6 mL) was added into a solution of compound **7-12** (1 eq) in THF (20 mL). The reaction mixture was heated and stirred at 95 °C for 18 hours. After that the mixture was allowed to cool down to room temperature and was concentrated under a reduced pressure. The residue was dissolved in 10 mL of H_2O and acidified with 1 M HCl. The white precipitate was filtered off and washed successively with MeOH to afford the acid intermediate **17a**-**g** without further purification.

Preparation of compound **17a**

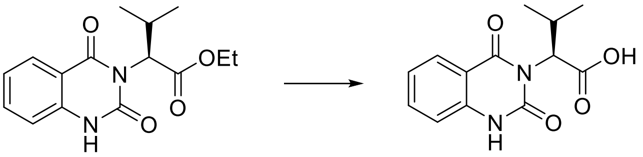

16a **17a**

(S)-2-(2,4-dioxo-1,4-dihydroquinazolin-3(2H)-yl)-3-methylbutanoic acid (**17a**): white solid, 1.26 g, 93% yield, m.p. >305.4 °C (decomposed). FTIR (cm^{-1}): 3250.1 (NH-C=O), 3078.9 (OH) 2970.3, 2924.8 and 2850.6 (=C-H aromatic), 1751 (O=C-OH), 1682.4 (O=C-N-R), and 1640.6 (O=C-NH). ^{1}H-NMR (400 MHz, DMSO-d_6) δ 12.61 (br(-OH), 1H), 11.61 (s(-NH), 1H), 7.95 (d, *J* = 7.7 Hz, 1H), 7.70 (td, *J* = 1.1, 7.7 Hz, 1H), 7.25 (d, *J* = 7.6 Hz, 1H), 7.22 (d, *J* = 8.0 Hz, 1H), 4.96 (d, *J* = 9.3 Hz, 1H), 2.66–2.57 (m, 1H) 1.17 (d, *J* = 6.4 Hz, 3H), 0.68 (d, *J* = 6.9 Hz, 3H). ^{13}C-NMR (100 MHz, DMSO-d_6) δ 172.21, 161.95, 150.22, 139.53, 135.19, 127.59, 122.64, 115,19, 113.48, 59.22, 26.93, 22.75, 19.28. ESI-HRMS (*m/z*): found 263.1025 $[M + H]^+$ (calcd. for $C_{13}H_{15}N_2O_4^+$ 263.1026).

Preparation of compound **17b**

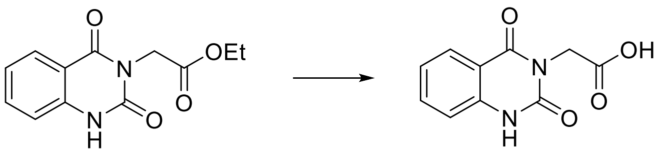

16b **17b**

2-(2,4-dioxo-1,4-dihydroquinazolin-3(2H)-yl)acetic acid (**17b**): white solid, 0.85 g, 86% yield. ^{1}H-NMR (400 MHz, DMSO-d$_6$) δ 11.57 (s, 1H, NH), 7.93 (dd, *J* = 7.9, 1.0 Hz, 1H), 7.68 (td, *J* = 7.7, 1.1 Hz, 1H), 7.25 – 7.19 (m, 2H), 4.52 (s, 2H). ^{13}C-NMR (101 MHz, DMSO-d$_6$) δ 169.49, 161.70, 149.96, 139.46, 135.38, 127.49, 122.82, 115.34, 113.53, 41.68. ESI-HRMS (*m/z*): found 243.0375 [M+Na]$^+$ (calcd for $C_{10}H_8O_4N_2Na^+$ 243.0376).

Preparation of compound **17c**

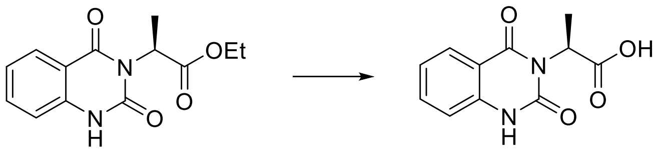

16c **17c**

(S)-2-(2,4-dioxo-1,4-dihydroquinazolin-3(2H)-yl)propanoic acid (**17c**): white solid, 0.44 g, 44% yield. ^{1}H-NMR (400 MHz, DMSO-d$_6$) δ 12.63 (s, 1H, O=C-OH), 11.55 (s, 1H, NH), 7.93 (d, *J* = 7.1 Hz, 1H), 7.68 (td, *J* = 7.7, 1.4 Hz, 1H), 7.23 (t, *J* = 7.1 Hz, 1H), 7.18 (d, *J* = 8.1 Hz, 1H), 5.41 (q, *J* = 6.9 Hz, 1H), 1.47 (d, *J* = 7.0 Hz, 3H). ^{13}C-NMR (101 MHz, DMSO-d$_6$) δ 171.36, 161.43, 149.59, 139.41, 135.36, 127.51, 122.79, 115.23, 113.62, 48.65, 14.32. ESI-HRMS (*m/z*): found 235.0709 [M+H]$^+$ (calcd for $C_{11}H_{11}N_2O_4^+$ 235.0713).

Preparation of compound **17d**

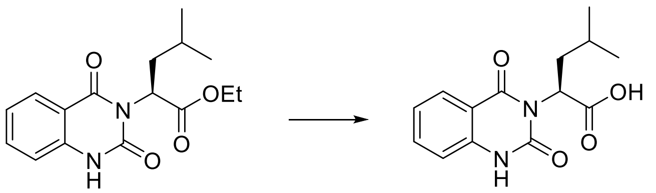

16d **17d**

(S)-2-(2,4-dioxo-1,4-dihydroquinazolin-3(2H)-yl)-4-methylpentanoic acid (**17d**): pale yellow solid, 2.06 g, 59% yield. ^{1}H-NMR (400 MHz, DMSO-d$_6$) δ 12.68 (s, 1H, O=C-OH), 11.56 (s, 1H, NH), 7.94 (d, *J* = 7.1 Hz, 1H), 7.69 (td, *J* = 7.7, 1.4 Hz, 1H), 7.24 (d, *J* = 7.4 Hz, 1H), 7.20 (d, *J* = 7.9 Hz, 1H), 5.40 (dd, *J* = 9.1, 5.1 Hz, 1H), 2.04 – 1.92 (m, 2H), 1.48 – 1.39 (m, 1H), 0.90 (d, *J* = 6.5 Hz, 3H), 0.84 (d, *J* = 6.6 Hz, 3H). ^{13}C-NMR (151 MHz, DMSO-d$_6$) δ 171.29, 161.80,

149.85, 139.44, 135.42, 127.61, 122.83, 115.24, 113.43, 60.74, 37.31, 23.05, 21.82, 13.98. ESI-HRMS (*m/z*): found 277.1177 [M+H]$^+$ (calcd for $C_{14}H_{17}N_2O_4^+$ 277.1183).

Preparation of compound **17e**

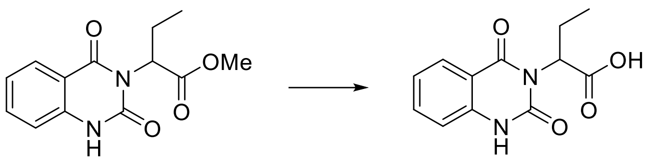

16e **17e**

2-(2,4-dioxo-1,4-dihydroquinazolin-3(2H)-yl)butanoic acid (**17e**): white solid, 2.10 g, quantitative yield. ^{1}H-NMR (400 MHz, DMSO-d$_6$) δ 11.44 (s, 1H, NH), 7.87 (d, *J* = 7.9 Hz, 1H), 7.58 (td, *J* = 7.7, 1.4 Hz, 1H), 7.19 (d, *J* = 8.2 Hz, 1H), 7.14 (td, *J* = 7.6, 0.6 Hz, 1H), 4.93 (dd, *J* = 10.2, 4.9 Hz, 1H), 2.25 – 2.15 (m, 1H), 2.14 – 2.03 (m, 1H), 0.74 (t, *J* = 7.5 Hz, 3H). ^{13}C-NMR (101 MHz, DMSO-d$_6$) δ 172.63, 162.28, 150.72, 139.77, 134.44, 127.40, 122.01, 114.96, 114.17, 58.05, 22.22, 11.98. ESI-HRMS (*m/z*): found 249.0872 [M+H]$^+$ (calcd for $C_{12}H_{13}N_2O_4^+$ 249.0870).

Preparation of compound **17f**

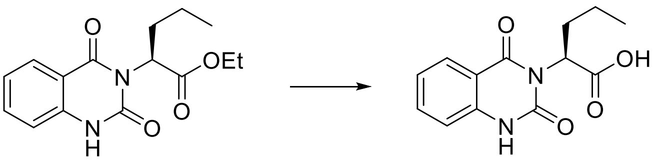

16f **17f**

(S)-2-(2,4-dioxo-1,4-dihydroquinazolin-3(2H)-yl)pentanoic acid (**17f**): yellow oil, 0.80 g, 24% yield. ^{1}H-NMR (600 MHz, DMSO-d$_6$) δ 11.62 (s, 1H, O=C-OH), 11.56 (s, 1H, NH), 7.93 (dd, *J* = 7.9, 1.4 Hz, 1H), 7.72 – 7.66 (m, 1H), 7.26 – 7.19 (m, 2H), 5.34 (dd, *J* = 9.2, 5.5 Hz, 1H), 2.10 – 1.99 (m, 2H), 1.29 – 1.15 (m, 2H), 0.85 (t, *J* = 7.3 Hz, 3H). ^{13}C-NMR (151 MHz, DMSO-d$_6$) δ 171.10, 169.49, 161.78, 149.85, 139.46, 135.40, 127.59, 122.82, 115.24, 60.65, 30.20, 19.17, 13.65. ESI-HRMS (*m/z*): found 263.1030 [M+H]$^+$ (calcd for $C_{13}H_{15}N_2O_4^+$ 263.1026).

Preparation of compound **17g**

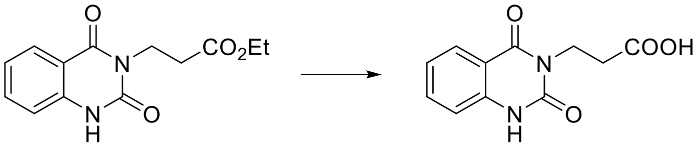

16g **17g**

3-(2,4-dioxo-1,4-dihydroquinazolin-3(2H)-yl)propanoic acid (**17g**): white solid, 4.31 g, 99% yield. ^{1}H-NMR (400 MHz, DMSO-d$_6$) δ 12.35 (s, 1H, O=C-OH), 11.44 (s, 1H, NH), 7.91 (dd, *J* = 7.8, 0.8 Hz, 1H), 7.64 (td, *J* = 7.7, 1.4 Hz, 1H), 7.22 – 7.15 (m, 2H), 4.10 (t, *J* = 7.7 Hz, 2H), 2.53 (t, *J* = 7.8 Hz, 2H). ^{13}C-NMR (101 MHz, DMSO-d$_6$) δ 172.48, 161.86, 150.00, 139.45, 135.03, 127.38, 122.54, 115.15, 113.79, 36.12, 32.12. ESI-HRMS (*m/z*): found 235.0711 $[M+H]^+$ (calcd for $C_{11}H_{11}N_2O_4^+$ 235.0713).

General procedure D; (Preparation of compound **2, 5-10** and **18-29**)

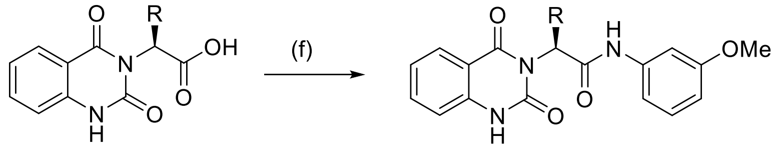

17a-f

17a: R = isopropyl
17b: R = H
17c: R = CH_3
17d: R = isobutyl
17e: R = *rac*-ethyl
17f: R = propyl

2 and 5-9

2: R = isopropyl
5: R = H
6: R = CH_3
7: R = isobutyl
8: R = *rac*-ethyl
9: R = propyl

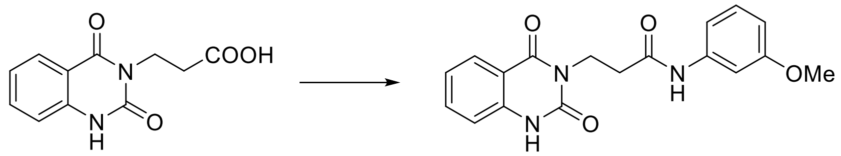

17g **10**

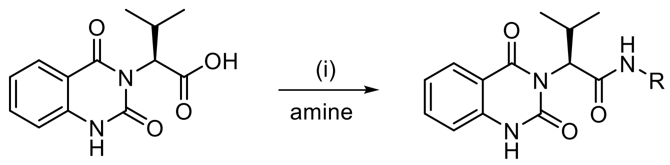

17a **18 - 29**

18: R = phenyl
19: R = 3-pyridyl
20: R = 4-pyridyl
21: R = 3-F-phenyl
22: R = 4-F-phenyl
23: R = 3,4-di-F-phenyl
24: R = 3-Cl-phenyl
25: R = 4-Cl-phenyl
26: R = 3,4-di-Cl-phenyl
27: R = 3-Cl-4-F-phenyl
28: R = benzyl
29: R = 4-MeO-phenyl

To a solution of acid **17a-17f** (1 eq) in DMF (4 mL), triethylamine (TEA) (1 eq) and HATU (1 eq) were added. The mixture was left stirring for 1 h at room temperature, after which amine (1.5 eq) was directly added and the reaction was left stirring at room temperature for 18 hours. After the reaction was completed, the solvent was removed under a reduced pressure. The residue was dissolved in EtOAc, and the solution was extracted with 0.4 M Na_2CO_3 solution and washed with water. The organic layer was collected, dried over $MgSO_4$ and evaporated under a reduced pressure. Purification was performed using CC over silica gel (10-50% EtOAc/Hexanes) or an automated flash column chromatography (Biotage®, gradient system of 10-50% EtOAc/Hexanes) or recrystallization with EtOAc to afford the desired quinazolinedione product **2, 5-10** and **18-29**.

Preparation of compound **2**

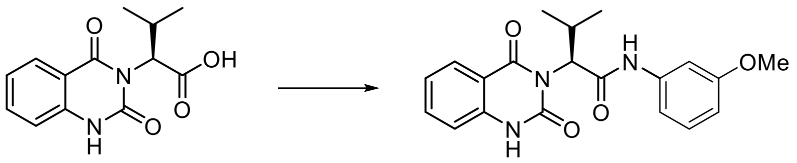

17a **2**

(S)-2-(2,4-dioxo-1,4-dihydroquinazolin-3(2H)-yl)-N-(3-methoxyphenyl)-3-methybutanamide (**1**): light brown solid, 0.29 g, 77% yield, m.p.: 72.8–73.4 °C. FTIR (cm^{-1}): 3206.5 and 3142.1 (NH-C=O), 2959.7, 2928.0 and 2872.3 (=C-H aromatic), 1715.7 and 1648.9 (O=C-N-H). ^{1}H-NMR (500 MHz, $CDCl_3$): δ = 10.51 (s, 1H), 8.87 (s, 1H), 8.06 (d, *J* = 10.0 Hz, 1H), 7.55 (td, *J* = 1.8, 9.5 Hz, 1H), 7.32 (s, 1H), 7.19 (td, *J* = 1.1, 9.6 Hz, 1H), 7.13 (t, *J* = 10.0 Hz, 2H), 6.98 (d, *J* = 10.0 Hz, 1H), 6.59 (dd, *J* = 2.34, 10.3 Hz, 1H), 5.30 (d, *J* = 13.4 Hz, 1H), 3.73 (s, 3H), 3.14-3.05 (m, 1H), 1.22 (d, *J* = 8.2 Hz, 3H), 0.85 (d, *J* = 8.4 Hz, 3H). ^{13}C-NMR (125 MHz, $CDCl_3$); δ = 167.36, 163.56, 160.07, 152.11, 139.08, 138.57, 135.76, 129.56, 123.75, 115.42, 114.08, 112.22, 110.26, 105.69, 64.37, 55.28, 26.80, 21.00, 19.10. ESI-HRMS (*m/z*): 368.1602 $[M + H]^+$ (calcd. for $C_{20}H_{22}N_3O_4^+$ 368.1602).

Preparation of compound **5**

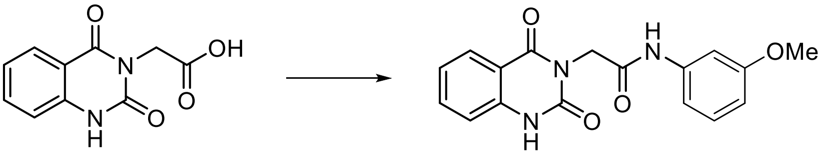

17b **5**

2-(2,4-dioxo-1,4-dihydroquinazolin-3(2H)-yl)-N-(3-methoxyphenyl)acetamide (**5**): white solid, 26.4 mg, 14% yield. ^{1}H NMR (400 MHz, DMSO-d_6) δ 11.57 (s, 1H), 10.30 (s, 1H), 7.94 (d, *J* = 7.3 Hz, 1H), 7.70 (td, *J* = 7.7, 1.2 Hz, 1H), 7.30-7.26 (m, 1H), 7.25 (d, *J* = 7.4 Hz, 1H), 7.22 (t, *J* = 7.3 Hz, 1H), 7.20 (t, *J* = 8.2 Hz, 1H), 7.08 (d, *J* = 8.1 Hz, 1H), 6.63 (dd, *J* = 8.2, 2.2 Hz, 1H), 4.70 (s, 2H), 3.71 (s, 3H). ^{13}C NMR (101 MHz, DMSO-d_6) δ 165.62, 161.95, 159.62, 150.16, 140.00, 139.54, 135.38, 129.70, 127.52, 122.82, 115.35, 113.66, 111.36, 109.03, 104.81, 55.02, 43.15. ESI-HRMS (*m/z*): 326.1130 $[M+H]^+$ (calcd for $C_{17}H_{16}N_3O_4^+$ 326.1135).

Preparation of compound **6**

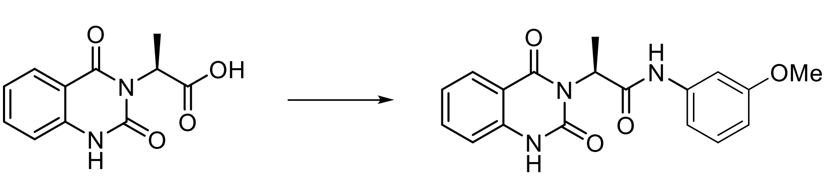

17c **7**

(S)-2-(2,4-dioxo-1,4-dihydroquinazolin-3(2H)-yl)-N-(3-methoxyphenyl)propanamide (**6**): white solid, 0.13 g, 38% yield. ^{1}H NMR (400 MHz, DMSO-d_6) δ 11.47 (s, 1H), 9.54 (s, 1H), 7.94 (d, *J* = 7.7 Hz, 1H), 7.67 (t, *J* = 7.5 Hz, 1H), 7.24 – 7.19 (m, 3H), 7.18 – 7.09 (m, 2H), 6.60 (d, *J* = 7.2 Hz, 1H), 5.44 (q, *J* = 6.5 Hz, 1H), 3.70 (s, 3H), 1.49 (d, *J* = 6.7 Hz, 3H). ^{13}C NMR (101 MHz, DMSO-d_6) δ 168.07, 161.85, 159.35, 149.77, 140.35, 139.63, 135.04, 129.16, 127.52, 122.47, 115.10, 114.34, 112.37, 108.77, 105.77, 54.98, 50.12, 14.25. ESI-HRMS (*m/z*): 340.1284 $[M+H]^+$ (calcd for $C_{18}H_{18}N_3O_4^+$ 340.1292).

Preparation of compound **7**

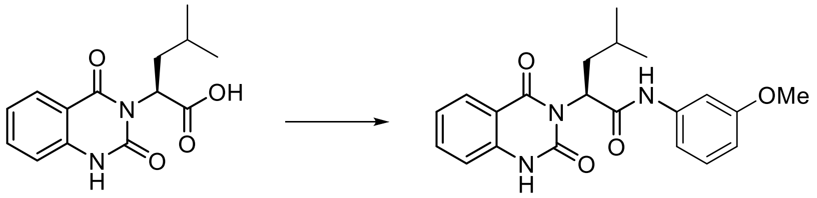

17d **7**

(S)-2-(2,4-dioxo-1,4-dihydroquinazolin-3(2H)-yl)-N-(3-methoxyphenyl)-4-methylpentanamide (**7**): pale yellow oil, 93.1 mg, 24% yield. ^{1}H NMR (400 MHz, DMSO-d_6) δ 11.48 (s, 1H), 9.51 (s, 1H), 7.95 (d, *J* = 7.8 Hz, 1H), 7.68 (td, *J* = 7.7, 1.3 Hz, 1H), 7.25 – 7.18 (m, 3H), 7.16 (t, *J* = 8.0 Hz, 1H), 7.11 (d, *J* = 8.2 Hz, 1H), 6.61 (dd, *J* = 7.9, 1.4 Hz, 1H), 5.44 (dd, *J* = 9.4, 4.8 Hz, 1H), 3.70 (s, 3H), 2.19 – 2.09 (m, 1H), 1.96 – 1.87 (m, 1H), 1.47 – 1.37 (m, 1H), 0.92 (d, *J* = 6.5 Hz, 3H), 0.86 (d, *J* = 6.6 Hz, 3H). ^{13}C NMR (151 MHz, DMSO) δ 167.99, 162.28, 159.32, 150.06, 140.17, 139.67, 135.09, 129.11, 127.62, 122.52, 115.13, 114.24, 112.74, 108.94, 106.13, 59.76, 54.99, 37.15, 24.85, 23.20, 21.93. ESI-HRMS (*m/z*): 382.1757 $[M+H]^+$ (calcd for $C_{21}H_{24}N_3O_4^+$ 382.1757).

Preparation of compound **8**

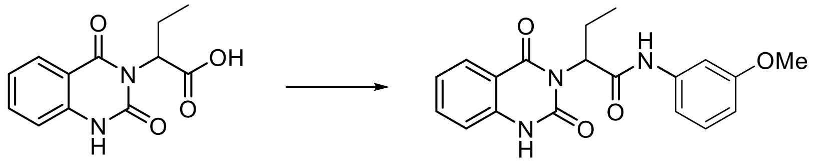

17e **8**

2-(2,4-dioxo-1,4-dihydroquinazolin-3(2H)-yl)-N-(3-methoxyphenyl)butanamide (**8**): pale yellow solid, 0.26 g, 73% yield. ^{1}H NMR (400 MHz, DMSO-d_6) δ 11.48 (s, 1H), 9.51 (s, 1H), 7.95 (dd, *J* = 9.0, 1.0 Hz, 1H), 7.68 (td, *J* = 7.7, 1.4 Hz, 1H), 7.25 – 7.20 (m, 3H), 7.18 – 7.11 (m, 2H), 6.63 – 6.58 (m, 1H), 5.33 (dd, *J* = 9.7, 5.1 Hz, 1H), 3.70 (s, 3H), 2.33 – 2.23 (m, 1H), 2.04 – 1.96 (m, 1H), 0.81 (t, *J* = 7.5 Hz, 3H). ^{13}C NMR (101 MHz, DMSO-d_6) δ 167.80, 162.31, 159.34, 150.13, 140.24, 139.71, 135.11, 129.15, 127.63, 122.53, 115.15, 114.17, 112.59, 108.86,

105.97, 55.70, 54.99, 21.09, 10.70. ESI-HRMS (*m/z*): 354.1445 [M+H]$^+$ (calcd for $C_{19}H_{20}N_3O_4^+$ 354.1448).

Preparation of compound **9**

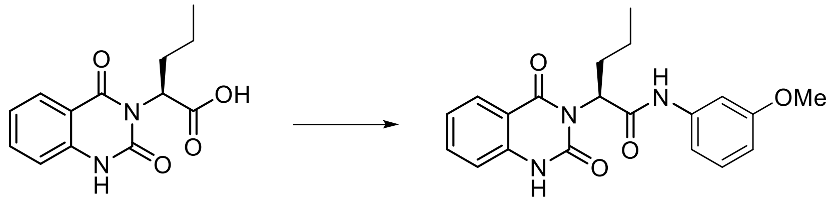

17f **9**

(S)-2-(2,4-dioxo-1,4-dihydroquinazolin-3(2H)-yl)-N-(3-methoxyphenyl)pentanamide (**9**): white solid, 0.19 g, 53% yield. ^{1}H NMR (400 MHz, DMSO-d$_6$) δ 11.48 (s, 1H), 9.51 (s, 1H), 7.94 (d, *J* = 7.9 Hz, 1H), 7.68 (td, *J* = 7.8, 1.2 Hz, 1H), 7.24 – 7.18 (m, 3H), 7.17 (d, *J* = 8.0 Hz, 1H), 7.14 – 7.10 (m, 1H), 6.63 – 6.58 (m, 1H), 5.40 (dd, *J* = 9.6, 4.9 Hz, 1H), 3.70 (s, 3H), 2.28 – 2.15 (m, 1H), 2.04 – 1.92 (m, 1H), 1.29 – 1.21 (m, 1H), 1.19 – 1.10 (m, 1H), 0.87 (t, *J* = 7.3 Hz, 3H). ^{13}C NMR (151 MHz, DMSO-d$_6$) δ 167.86, 162.25, 159.32, 150.07, 140.22, 139.68, 135.08, 129.11, 127.61, 122.50, 115.12, 114.18, 112.63, 108.86, 106.03, 54.98, 54.14, 30.03, 19.15, 13.77. ESI-HRMS (*m/z*): 368.1603 [M+H]$^+$ (calcd for $C_{20}H_{22}N_3O_4^+$ 368.1605).

Preparation of compound **10**

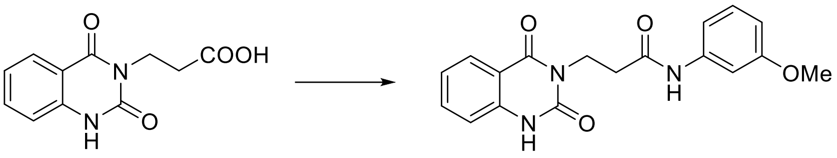

17g **10**

3-(2,4-dioxo-1,4-dihydroquinazolin-3(2H)-yl)-N-(3-methoxyphenyl)propenamide (**10**): white solid, 0.13 g, 38% yield. ^{1}H NMR (400 MHz, DMSO-d$_6$) δ 11.46 (s, 1H), 9.98 (s, 1H), 7.93 (dd, *J* = 7.9, 1.0 Hz, 1H), 7.65 (td, *J* = 7.7, 1.4 Hz, 1H), 7.29-7.23 (m, 1H), 7.21 (t, *J* = 7.7 Hz, 1H), 7.17 (d, *J* = 8.1 Hz, 2H), 7.08 (d, *J* = 8.3 Hz, 1H), 6.61 (dd, *J* = 8.1, 1.9 Hz, 1H), 4.20 (t, *J* = 7.4 Hz, 2H), 3.71 (s, 3H), 2.63 (t, *J* = 7.4 Hz, 2H). ^{13}C NMR (101 MHz, DMSO-d$_6$) δ 168.98, 161.88, 159.46, 150.06, 140.27, 139.44, 134.98, 129.42, 127.37, 122.49, 115.11, 113.82, 111.60, 108.57, 105.16, 54.94, 36.73, 34.67. ESI-HRMS (*m/z*): 340.1294 [M+H]$^+$ (calcd for $C_{18}H_{18}N_3O_4^+$ 340.1292).

Preparation of compound **18**

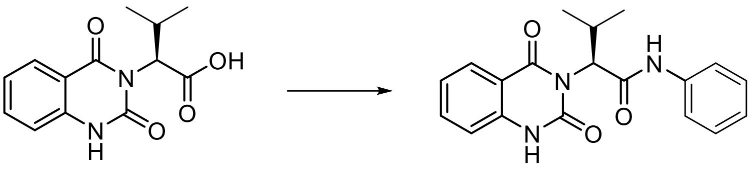

(S)-2-(2,4-dioxo-1,4-dihydroquinazolin-3(2H)-yl)-3-methyl-N-phenylbutanamide (**18**): white solid, 0.27 g, 78% yield. ^{1}H NMR (600 MHz, $CDCl_3$) δ 10.44 (s, 1H), 8.86 (s, 1H), 8.09 (t, *J* = 7.8 Hz, 1H), 7.59 – 7.54 (m, 1H), 7.53 (d, *J* = 7.9 Hz, 2H), 7.25 – 7.23 (m, 2H), 7.21 (td, *J* = 8.0, 0.8 Hz, 1H), 7.13 (t, *J* = 7.8 Hz, 1H), 7.04 (t, *J* = 7.4 Hz, 1H), 5.31 (d, *J* = 10.8 Hz, 1H), 3.17-3.07 (m, 1H), 1.22 (d, *J* = 6.5 Hz, 3H), 0.86 (d, *J* = 6.7 Hz, 3H). ^{13}C NMR (151 MHz, $CDCl_3$) δ 167.41, 163.70, 152.24, 138.70, 137.97, 135.87, 129.04, 128.71, 124.43, 123.88, 120.21, 115.50, 114.24, 26.94, 21.07, 21.01, 19.25. ESI-HRMS (*m/z*): 338.1494 [M+H]$^+$ (calcd for $C_{19}H_{20}N_3O_3^+$ 338.1499).

Preparation of compound **19**

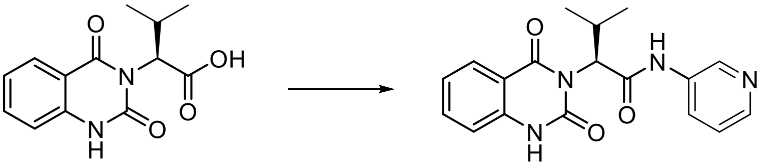

(S)-2-(2,4-dioxo-1,4-dihydroquinazolin-3(2H)-yl)-3-methyl-N-(pyridin-3-yl)butanamide (**19**): off-white solid, 0.19 g, 66% yield. ^{1}H NMR (600 MHz, DMSO-d_6) δ 11.54 (s, 1H), 9.71 (s, 1H), 8.65 (d, *J* = 2.4 Hz, 1H), 8.26 – 8.17 (m, 1H), 7.96 (d, *J* = 7.9 Hz, 1H), 7.95 – 7.92 (m, 1H), 7.69 (t, *J* = 7.7 Hz, 1H), 7.30 (dd, *J* = 8.3, 4.7 Hz, 1H), 7.24 (d, *J* = 8.9 Hz, 1H), 7.21 (d, *J* = 8.8 Hz, 1H), 5.07 (d, *J* = 8.8 Hz, 1H), 2.78 – 2.68 (m, 1H), 1.16 (d, *J* = 6.5 Hz, 3H), 0.69 (d, *J* = 7.0 Hz, 3H). ^{13}C NMR (151 MHz, DMSO-d_6) δ 167.97, 162.36, 150.23, 144.18, 142.02, 139.61, 135.68, 135.25, 127.73, 127.51, 123.34, 122.63, 115.19, 113.92, 58.77, 26.58, 22.41, 18.47. ESI-HRMS (*m/z*): 339.1452 [M+H]$^+$ (calcd for $C_{18}H_{19}N_4O_3^+$ 339.1452).

Preparation of compound **20**

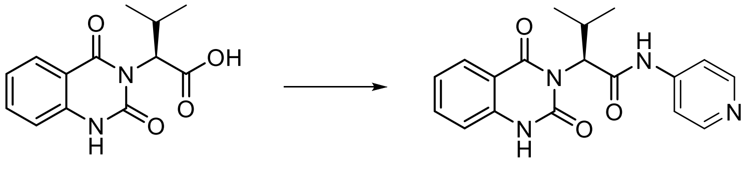

(S)-2-(2,4-dioxo-1,4-dihydroquinazolin-3(2H)-yl)-3-methyl-N-(pyridin-4-yl)butanamide (**20**): white solid, 0.13 g, 36% yield. ^{1}H NMR (600 MHz, DMSO-d_6) δ 11.55 (s, 1H), 9.88 (s, 1H),

8.36 (d, J = 6.4 Hz, 2H), 7.95 (d, J = 7.5 Hz, 1H), 7.69 (td, J = 8.0, 1.5 Hz, 1H), 7.55 (d, J = 6.4 Hz, 2H), 7.23 (d, J = 7.5 Hz, 1H), 7.22 (d, J = 7.8 Hz, 1H), 5.05 (d, J = 8.7 Hz, 1H), 2.76 – 2.66 (m, 1H), 1.15 (d, J = 6.5 Hz, 3H), 0.69 (d, J = 7.0 Hz, 3H). ^{13}C NMR (151 MHz, DMSO-d$_6$) δ 168.37, 162.34, 150.22, 150.07, 145.92, 139.60, 135.31, 127.74, 122.68, 115.23, 113.92, 113.85, 58.97, 26.64, 22.27, 18.40. ESI-HRMS (*m/z*): 339.1449 [M+H]$^+$ (calcd for $C_{18}H_{19}N_4O_3^+$ 339.1452).

Preparation of compound **21**

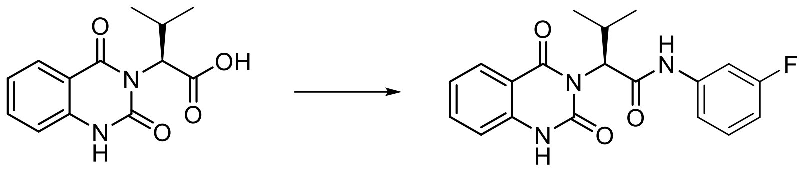

17e **21**

(S)-2-(2,4-dioxo-1,4-dihydroquinazolin-3(2H)-yl)-N-(3-fluorophenyl)-3-methylbutan-amide (**21**): pale yellow solid, 0.26 g, 72% yield. ^{1}H NMR (600 MHz, $CDCl_3$) δ 10.41 (s, 1H), 9.02 (s, 1H), 8.06 (d, J = 7.9 Hz, 1H), 7.58 (td, J = 7.7, 1.3 Hz, 1H), 7.52 (dd, J = 9.0, 2.0 Hz, 1H), 7.24 – 7.16 (m, 2H), 7.16 – 7.10 (m, 2H), 6.78 – 6.64 (m, 1H), 5.30 (d, J = 10.6 Hz, 1H), 3.14 – 3.04 (m, 1H), 1.21 (d, J = 6.5 Hz, 3H), 0.84 (d, J = 6.7 Hz, 3H). ^{13}C NMR (151 MHz, $CDCl_3$) δ 167.57, 163.76, 162.23, 152.19, 139.50, 138.64, 135.99, 130.09, 128.70, 124.00, 115.50, 115.41, 114.16, 111.00, 107.73, 107.56, 26.90, 21.03, 19.18. ESI-HRMS (*m/z*): 356.1399 [M+H]$^+$ (calcd for $C_{19}H_{19}FN_3O_3^+$ 356.1405).

Preparation of compound **22**

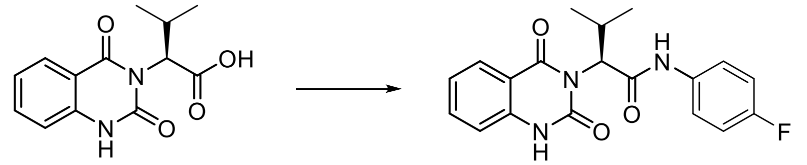

17e **22**

(S)-2-(2,4-dioxo-1,4-dihydroquinazolin-3(2H)-yl)-N-(4-fluorophenyl)-3-methylbutan-amide (**22**): white solid, 0.21 g, 36% yield. ^{1}H NMR (600 MHz, $CDCl_3$) δ 10.41 (s, 1H), 8.94 (s, 1H), 8.06 (d, J = 7.7 Hz, 1H), 7.56 (td, J = 7.9, 1.7 Hz, 1H), 7.48 (dd, J = 8.9, 4.8 Hz, 2H), 7.20 (td, J = 7.7, 0.7 Hz, 1H), 7.13 (t, J = 7.4 Hz, 1H), 6.93 (t, J = 8.6 Hz, 2H), 5.29 (d, J = 10.7 Hz, 1H), 3.18 – 2.98 (m, 1H), 1.20 (d, J = 6.6 Hz, 3H), 0.84 (d, J = 6.7 Hz, 3H). ^{13}C NMR (151 MHz, $CDCl_3$) δ 167.49, 163.73, 160.26, 158.65, 152.16, 138.70, 135.88, 133.98, 128.69, 123.91, 122.03, 121.98, 115.70, 115.55, 115.46, 114.22, 26.95, 21.06, 19.22. ESI-HRMS (*m/z*): 356.1415 [M+H]$^+$ (calcd for $C_{19}H_{19}FN_3O_3^+$ 356.1405).

Preparation of compound **23**

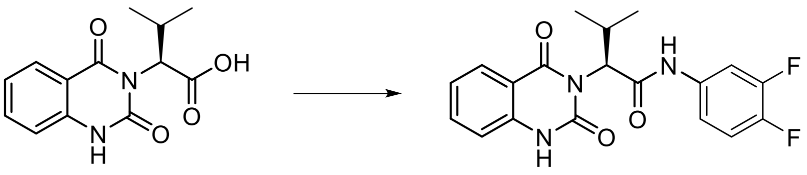

17e **23**

(S)-N-(3,4-difluorophenyl)-2-(2,4-dioxo-1,4-dihydroquinazolin-3(2H)-yl)-3-methylbut-anamide (**23**): pale yellow solid, 0.18 g, 46% yield. ^{1}H NMR (600 MHz, $CDCl_3$) δ 10.33 (s, 1H), 9.07 (s, 1H), 8.06 (d, *J* = 7.2 Hz, 1H), 7.65 – 7.61 (m, 1H), 7.59 (td, *J* = 7.7, 1.4 Hz, 1H), 7.21 (t, *J* = 7.4 Hz, 1H), 7.12 (d, *J* = 8.1 Hz, 1H), 7.11 – 7.06 (m, 1H), 7.04 – 6.97 (m, 1H), 5.29 (d, *J* = 10.6 Hz, 1H), 3.11 – 3.04 (m, 1H), 1.19 (d, *J* = 6.5 Hz, 3H), 0.84 (d, *J* = 6.7 Hz, 3H). ^{13}C NMR (151 MHz, $CDCl_3$) δ 167.61, 163.78, 152.11, 150.96, 149.24, 146.16, 138.63, 136.02, 134.49, 128.71, 124.05, 117.23, 115.81, 115.45, 114.16, 110.02, 26.92, 20.99, 19.16. ESI-HRMS (*m/z*): 374.1302 $[M+H]^+$ (calcd for $C_{19}H_{17}F_2N_3O_3^+$ 374.1311).

Preparation of compound **24**

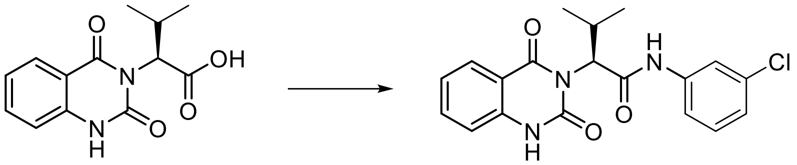

17e **24**

(S)-N-(3-chlorophenyl)-2-(2,4-dioxo-1,4-dihydroquinazolin-3(2H)-yl)-3-methylbutana-mide (**24**): pale yellow solid, 0.22 g, 69% yield. ^{1}H NMR (400 MHz, DMSO-d_6) δ 11.53 (s, 1H), 9.68 (s, 1H), 7.95 (d, *J* = 7.8 Hz, 1H), 7.69 (td, *J* = 7.7, 1.3 Hz, 2H), 7.46 (d, *J* = 8.2 Hz, 1H), 7.28 (t, *J* = 8.1 Hz, 1H), 7.23 (t, *J* = 8.8 Hz, 2H), 7.07 (dd, *J* = 7.9, 1.3 Hz, 1H), 5.03 (d, *J* = 8.8 Hz, 1H), 2.77 – 2.64 (m, 1H), 1.15 (d, *J* = 6.5 Hz, 3H), 0.68 (d, *J* = 7.0 Hz, 3H). ^{13}C NMR (151 MHz, $CDCl_3$) δ 167.61, 163.74, 152.19, 139.16, 138.63, 136.02, 134.66, 129.99, 128.71, 124.41, 124.00, 120.26, 118.14, 115.48, 114.21, 114.16, 26.91, 21.05, 19.19. ESI-HRMS (*m/z*): 372.1116 $[M+H]^+$ (calcd for $C_{19}H_{19}ClN_3O_3^+$ 372.1109).

Preparation of compound **25**

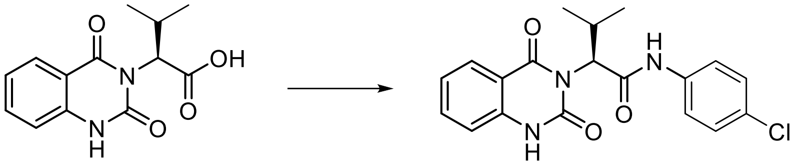

17e **25**

(S)-N-(4-chlorophenyl)-2-(2,4-dioxo-1,4-dihydroquinazolin-3(2H)-yl)-3-methylbutanamide (**25**): white solid, 0.17 g, 44% yield. ^{1}H NMR (400 MHz, DMSO-d_6) δ 11.52 (s, 1H), 9.64 (s, 1H), 7.95 (d, J = 7.8 Hz, 1H), 7.69 (td, J = 7.7, 1.3 Hz, 1H), 7.54 (d, J = 8.9 Hz, 2H), 7.31 (d, J = 8.9 Hz, 2H), 7.22 (t, J = 8.9 Hz, 2H), 5.02 (d, J = 8.8 Hz, 1H), 2.79 – 2.64 (m, 1H), 1.15 (d, J = 6.5 Hz, 3H), 0.67 (d, J = 7.0 Hz, 3H). ^{13}C NMR (151 MHz, $CDCl_3$) δ 167.40, 163.68, 151.99, 138.55, 136.60, 135.97, 131.04, 129.89, 129.35, 129.04, 128.91, 125.95, 124.05, 121.39, 115.29, 114.28, 26.92, 19.24, 14.26. ESI-HRMS (*m/z*): 372.1108 $[M+H]^+$ (calcd for $C_{19}H_{19}ClN_3O_3^+$ 372.1109).

Preparation of compound **26**

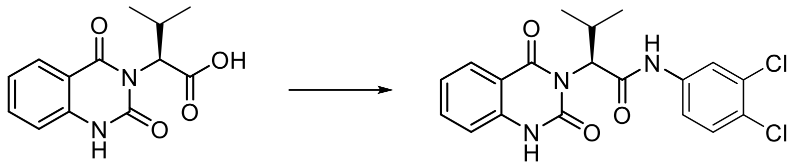

17e **26**

(S)-N-(3,4-dichlorophenyl)-2-(2,4-dioxo-1,4-dihydroquinazolin-3(2H)-yl)-3-methylbutanamide (**26**): white solid, 0.17 g, 43% yield. ^{1}H NMR (400 MHz, DMSO-d_6) δ 11.55 (s, 1H), 9.77 (s, 1H), 7.95 (d, J = 7.8 Hz, 1H), 7.88 (s, 1H), 7.70 (td, J = 7.7, 1.3 Hz, 1H), 7.51 (s, 2H), 7.25 – 7.20 (m, 2H), 5.04 (d, J = 8.7 Hz, 1H), 2.77 – 2.64 (m, 1H), 1.15 (d, J = 6.5 Hz, 3H), 0.68 (d, J = 7.0 Hz, 3H). ^{13}C NMR (151 MHz, DMSO-d_6) δ 167.87, 162.34, 150.20, 139.58, 139.25, 135.29, 130.66, 130.35, 127.73, 124.59, 122.68, 121.40, 120.19, 115.21, 113.86, 58.81, 26.59, 22.34, 18.43. ESI-HRMS (*m/z*): 406.0719 $[M+H]^+$ (calcd for $C_{19}H_{18}Cl_2N_3O_3^+$ 406.0720).

Preparation of compound **27**

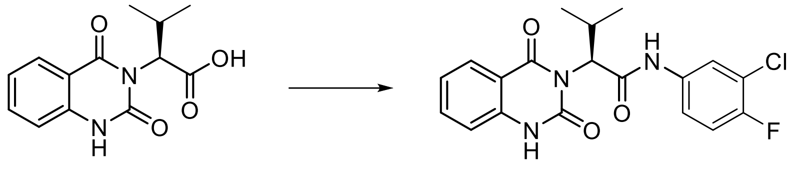

17e **27**

(S)-N-(3-chloro-4-fluorophenyl)-2-(2,4-dioxo-1,4-dihydroquinazolin-3(2H)-yl)-3-methylbutanamide (**27**): yellow oil, 0.14 g, 34% yield. ^{1}H NMR (400 MHz, DMSO-d_6) δ 11.54 (s, 1H), 9.69 (s, 1H), 7.95 (d, J = 7.5 Hz, 1H), 7.79 (dd, J = 6.9, 2.5 Hz, 1H), 7.69 (td, J = 7.7, 1.3 Hz, 1H), 7.50 – 7.43 (m, 1H), 7.31 (t, J = 9.1 Hz, 1H), 7.26 – 7.20 (m, 2H), 5.03 (d, J = 8.8 Hz, 1H), 2.76 – 2.65 (m, 1H), 1.15 (d, J = 6.4 Hz, 3H), 0.67 (d, J = 7.0 Hz, 3H). ^{13}C NMR (151 MHz, DMSO-d_6) δ 170.34, 167.70, 162.34, 139.59, 136.31, 136.29, 135.25, 127.72, 122.64, 121.77, 120.56, 118.83, 116.61, 115.19, 113.88, 58.75, 26.55, 22.37, 18.45. ESI-HRMS (*m/z*): 390.1016 $[M+H]^+$ (calcd for $C_{19}H_{18}ClFN_3O_3^+$ 390.1015).

Preparation of compound **28**

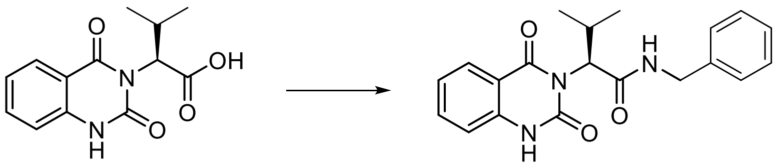

17e **28**

(S)-N-benzyl-2-(2,4-dioxo-1,4-dihydroquinazolin-3(2H)-yl)-3-methylbutanamide (**28**): white solid, 0.14 g, 37% yield. ^{1}H NMR (400 MHz, DMSO-d$_6$) δ 11.50 (s, 1H), 8.31 (t, *J* = 5.9 Hz, 1H), 7.94 (d, *J* = 7.9 Hz, 1H), 7.66 (td, *J* = 7.7, 1.3 Hz, 1H), 7.28 – 7.23 (m, 2H), 7.22 – 7.14 (m, 5H), 4.94 (d, *J* = 9.2 Hz, 1H), 4.31 – 4.15 (m, 2H), 2.79 – 2.64 (m, 1H), 1.16 (d, *J* = 6.4 Hz, 3H), 0.64 (d, *J* = 6.9 Hz, 3H). ^{13}C NMR (151 MHz, DMSO) δ 168.61, 162.32, 150.22, 139.91, 139.66, 135.04, 128.03, 127.64, 126.97, 126.44, 122.48, 115.05, 114.07, 58.69, 42.12, 26.37, 22.88, 18.74. ESI-HRMS (*m/z*): 352.1651 [M+H]$^+$ (calcd for $C_{20}H_{22}N_3O_3^+$ 352.1656).

Preparation of compound **29**

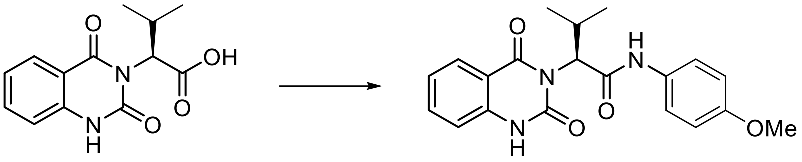

17e **29**

(S)-2-(2,4-dioxo-1,4-dihydroquinazolin-3(2H)-yl)-N-(4-methoxyphenyl)-3-methylbuta-namide (**29**): white solid, 0.14 g, 36% yield. ^{1}H NMR (400 MHz, DMSO-d$_6$) δ 11.49 (s, 1H), 9.39 (s, 1H), 7.95 (d, *J* = 7.7 Hz, 1H), 7.68 (td, *J* = 7.7, 1.3 Hz, 1H), 7.37 (d, *J* = 9.0 Hz, 2H), 7.22 (t, *J* = 8.9 Hz, 2H), 6.83 (d, *J* = 9.0 Hz, 2H), 5.00 (d, *J* = 8.9 Hz, 1H), 2.78 – 2.67 (m, 1H), 1.15 (d, *J* = 6.5 Hz, 3H), 0.67 (d, *J* = 7.0 Hz, 3H). ^{13}C NMR (151 MHz, DMSO-d$_6$) δ 167.07, 162.36, 155.32, 150.28, 139.63, 135.11, 132.04, 127.69, 122.53, 122.35, 115.11, 114.04, 113.47, 58.86, 55.14, 26.49, 22.59, 18.56. ESI-HRMS (*m/z*): 368.1612 [M+H]$^+$ (calcd for $C_{20}H_{22}N_3O_4^+$ 368.1605).
